# The kinetoplastid kinetochore protein KKT23 acetyltransferase is a structural homolog of GCN5 that acetylates the histone H2A C-terminal tail

**DOI:** 10.1016/j.str.2024.10.031

**Published:** 2024-11-22

**Authors:** Patryk Ludzia, Midori Ishii, Gauri Deák, Christos Spanos, Marcus D. Wilson, Christina Redfield, Bungo Akiyoshi

**Affiliations:** 1 Department of Biochemistry, University of Oxford, Oxford OX1 3QU, UK; 2 Wellcome Centre for Cell Biology, University of Edinburgh, Edinburgh EH9 3BF, UK

## Abstract

The kinetochore is the macromolecular protein machine that drives chromosome segregation in eukaryotes. In an evolutionarily divergent group of organisms called kinetoplastids, kinetochores are built using a unique set of proteins (KKT1–25 and KKIP1–12). KKT23 is a constitutively localized kinetochore protein containing a C-terminal acetyltransferase domain of unknown function. Here, using X-ray crystallography and nuclear magnetic resonance (NMR) spectroscopy, we have determined the structure and dynamics of the KKT23 acetyl-transferase domain from *Trypanosoma brucei* and found that it is structurally similar to the GCN5 histone acetyltransferase domain. We find that KKT23 can acetylate the C-terminal tail of histone H2A and that knockdown of KKT23 results in decreased H2A acetylation levels in *T. brucei*. Finally, we have determined the crystal structure of the N-terminal region of KKT23 and shown that it interacts with KKT22. Our study provides important insights into the structure and function of the unique kinetochore acetyltransferase in trypanosomes.

## Graphical abstract

**Graphical abstract:**
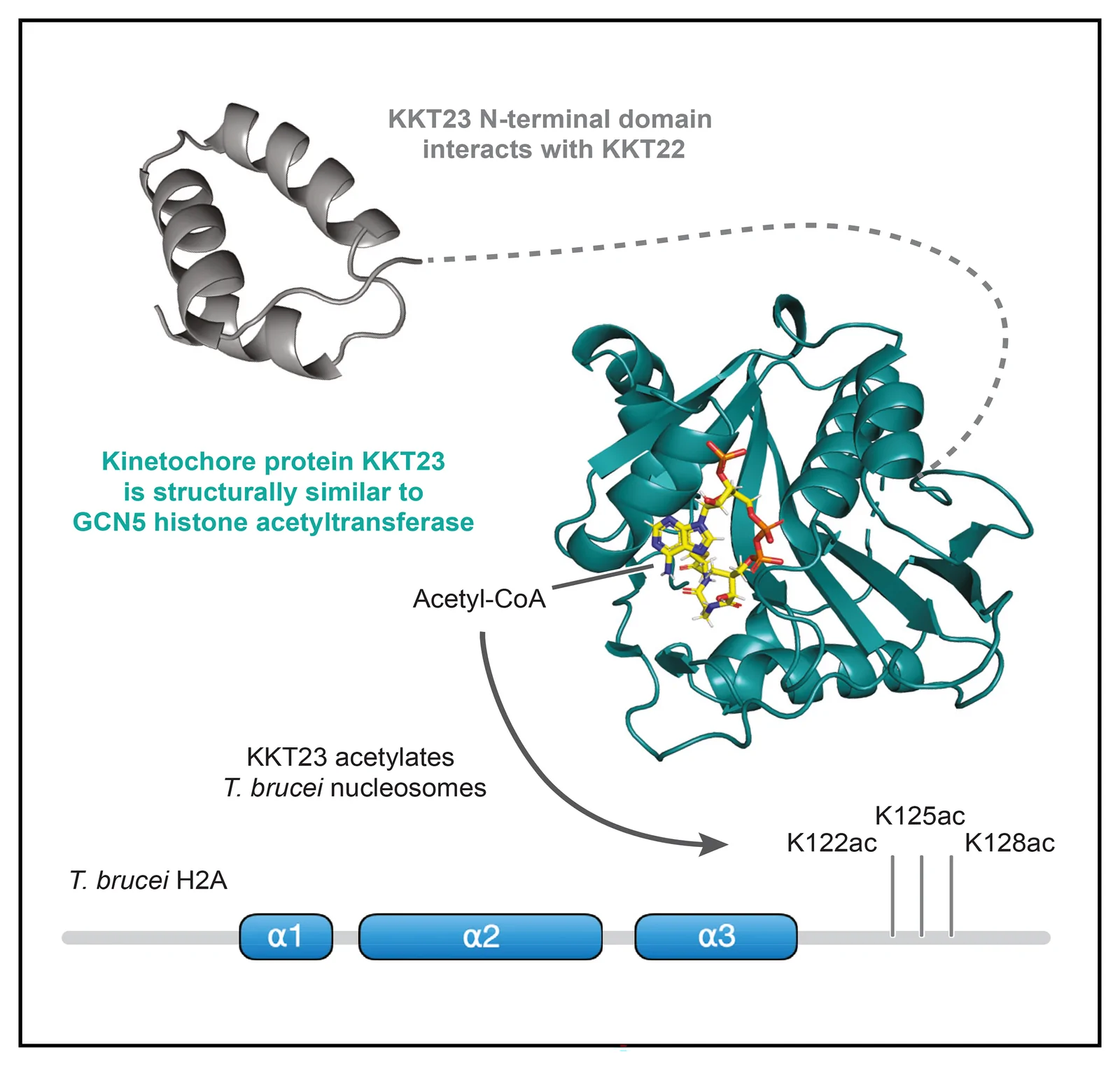

## Introduction

**Figure 1:**
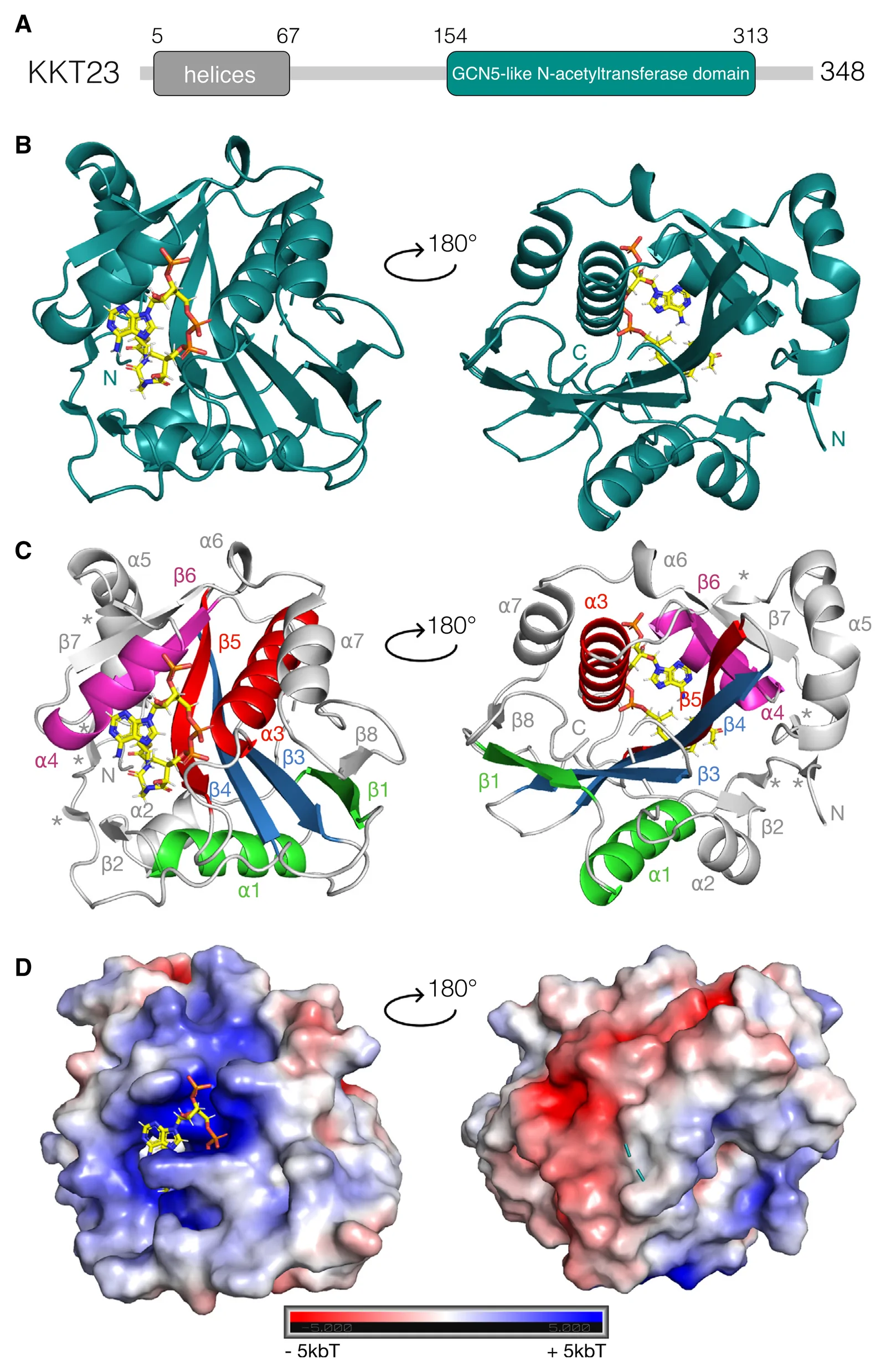
Crystal structure of KKT23^125–348^ bound to acetyl coenzyme A (A)Schematic representation of the KKT23 sequence. The N-terminal region of KKT23 is predicted to be mostly helical, while the C-terminal region is predicted to have a GCN5-related N-acetyltransferase domain fold.^6^ See also Figure S1. (B) Ribbon model of the *Trypanosoma brucei* KKT23^125−348^ crystal structure. Acetyl coenzyme A, shown as sticks is colored by the elements (C - yellow, N - blue, O - red, P- orange). The secondary structure elements are labeled in (C). N and C represent the N- and C-termini, respectively. (C) Motifs of the GNAT domain are highlighted in the crystal structure using different colors: motif A (red), B (magenta), C (green), and D (blue). The core of the enzyme is composed of motifs A and D. The four asterisks represent short β-strands, which were not included in the numbering of α/β elements. See also Figure S4 and Data S1. (D) The surface electrostatic potential is colored from red (acidic) to blue (basic), −5 kbT to +5 kbT, as calculated by APBS electrostatic plugin in Pymol.^10^ See also Data S1.

During cell division, genetic material must be replicated and segregated through the process of chromosome segregation.^1^ This process is facilitated by the kinetochore complex, whose components are widely conserved across eukaryotes.^2,3^ However, a unicellular group of eukaryotes called kinetoplastids has a unique set of kinetochore proteins that lack a significant similarity to conventional kinetochore proteins. These proteins were discovered in *Trypanosoma brucei* and consist of kineto-plastid kinetochore proteins (KKT1–25) and KKT-interacting proteins (KKIP1–12).^4–8^ In this study, we focus on KKT23, which was identified as a protein that co-purified with a constitutively localized kinetochore protein KKT3.^6^ Fluorescence microscopy of YFP-KKT23 revealed that it too localizes at the kinetochore throughout the cell cycle, and its immunoprecipitation from trypanosomes revealed co-purification of many kinetochore proteins, including KKT3 and KKT22.^6^ An RNAi-mediated knockdown of KKT23 resulted in growth defect, showing that KKT23 is a vital component of the kinetochore complex in kinetoplastids.^9^ Intriguingly, KKT23 has a predicted GCN5-related N-acetyltransferase (GNAT) domain in its C-terminal region (Figure 1A) that is highly conserved among kinetoplastids.^6^ Although there are acetyltransferases that regulate kinetochore functions in humans (e.g., MYST family acetyltransferases called TIP60 and KAT7), they are not considered as structural components of the kinetochore complex.^11,12^ Therefore, the presence of a GNAT protein is a unique feature of kinetochores in kinetoplastids. The function or structure of KKT23 remains unknown.

In this report, we present structural and functional analysis of KKT23 in *T. brucei*. We have revealed that the structure of its acetyltransferase domain is most similar to the GCN5 histone acetyltransferase. We also show that KKT23 can acetylate nucleosomes *in vitro* and have identified specific sites on histone H2A that are acetylated by KKT23. Notably, we have found that knockdown of KKT23 results in reduced acetylation levels on histone H2A in *T. brucei*. Our study has also revealed that KKT23 forms a complex with other kinetochore proteins, including KKT3 and KKT22, via its N-terminal region. These results provide valuable insights into the structure and function of the KKT23 acetyltransferase located at kinetoplastid kinetochores.

## Results

### C-terminal region of KKT23 has an acetyltransferase domain

Our sequence analysis of KKT23 predicted a helical region near the N-terminus (5–67), followed by a stretch of unstructured residues (72–126) (Figures S1A and S1B). The C-terminal region of KKT23 (129–348) is predicted to be a mixture of α-helices and β-strands, consistent with the GNAT domain prediction (residues 154–313) in that region (Figures 1A and S1A).^6^ We first employed size exclusion chromatography with multi-angle light scattering (SEC-MALS) and analytical ultracentrifugation (AUC) and found that the recombinant full-length protein is monomeric (Figure S2). To determine a high-resolution structure of KKT23, we next used X-ray crystallography. However, no crystal for the full-length protein was obtained. KKT23^125−348^ gives a well-resolved NMR spectrum characteristic of a stable folded protein domain (Figure S3). Addition of acetyl coenzyme A (acetyl-CoA), a known cofactor of acetyltransferases, leads to changes in the NMR spectrum of KKT23, which indicate formation of a KKT23-acetyl-CoA complex (Figure S3). We therefore aimed to solve the structure of the acetyltransferase domain (KKT23^125−348^) in the presence of acetyl-CoA. These efforts led to robust crystal formation, with the best crystals diffracting up to 1.8 Å resolution. Our attempts to solve the native structure using molecular replacement were unsuccessful. Instead, the X-ray structure of native KKT23^125−348^ was solved to 1.8 Å resolution using a sele-nomethionine derivative of KKT23^125−348^ (Figure 1B). The data collection and refinement statistics of both structures are summarized in Table 1.

In the asymmetric unit of the crystal, two molecules (A and B) of KKT23^125−348^ are both bound to acetyl-CoA. In molecule A, the electron density is missing for the N-terminal residue Q125 and residues 192–200, whereas in molecule B, the N-terminal regions 125–126, 137–139, and 195–198 are missing, most likely because they are disordered and/or flexible. The overall structure of KKT23^125−348^ has a mixed α/β topology containing 7 α-helices and 8 β-strands organized in 4 motifs (A, B, C, and D), as shown in Figure 1C and Data S1; this motif organization is consistent with previously reported structures of N-acetyltransferases.^13^ The core of the enzyme is built by a three-stranded antiparallel β-sheet (strands 3, 4, and 5) that sits below α-helix 3 (motifs A and D). The β-strands β3 and β4 (motif D) near the N-terminus are hydrogen-bonded to each other, most likely stabilizing the core region. Another part of the protein core is a β-loop-α motif built by β6 and α4 (motif B). In contrast to the secondary structure prediction (Figure S1A), no helices are observed in the crystal structure between residues 125 and 150. Using DSSP Stride,^14^ additional short β-strands were found in the structure. Two of these strands, involving residues 129–130 and 133–134, are located in the N-terminal region. The strand between residues 129 and 130 makes contacts with region 308–309, while region 133–134 is hydrogen bonded to β2 as shown in Figures 1C and S4. The hydrogen bonds formed by these short strands stabilize the mostly unstructured N-terminal region, which explains its well-defined electron density. Finally, the overall electrostatic potential of the protein surface shows a mixture of basic and acidic regions with the most positively charged region binding to acetyl-CoA (Figure 1D and Data S1).

### Acetyl-CoA is coordinated by residues located in motif A of the KKT23 GNAT domain

**Figure 2:**
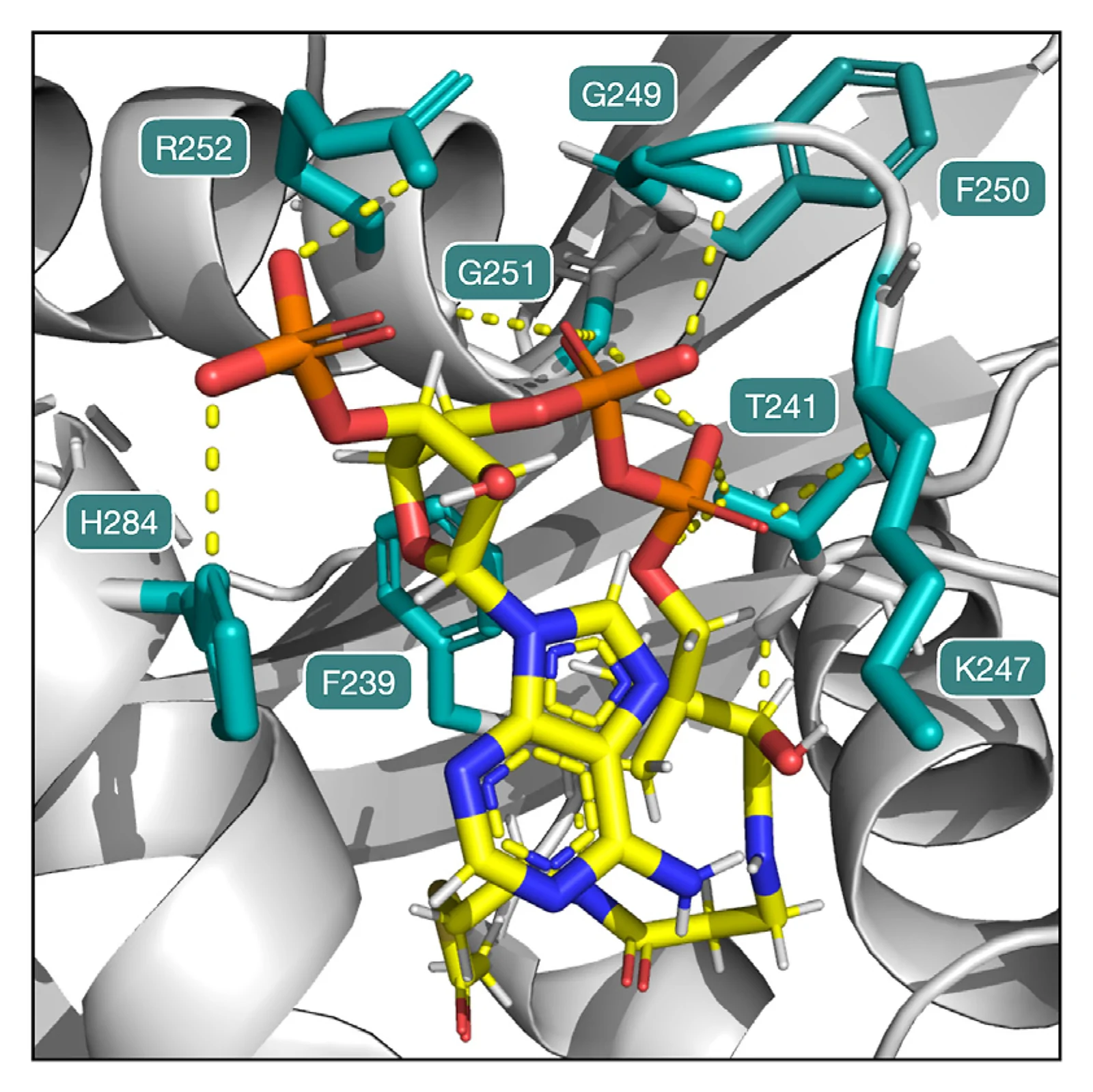
Hydrogen bonds coordinate acetyl-CoA in the KKT23^125–348^ crystal structure Residues within the KKT23^125−348^ crystal structure (teal) that are coordinating acetyl coenzyme A using hydrogen bonds are shown. Acetyl coenzyme A is colored by the elements and hydrogen bonds are shown as yellow dotted lines. See also Figure S5.

Based on the analysis using PDBePISA^15^ and the crystal structure, acetyl-CoA is coordinated by neighboring residues through both hydrogen bonds and hydrophobic interactions (Figures 2 and S5). Residues that were found to form hydrogen bonds with acetyl-CoA in both molecules of the asymmetric unit include F239, T241, K247, G249, F250, G251, R252, and H284. In molecule B, additional hydrogen bonds were observed with H248 and R333. F239 and T241 are located on β5; K247 occupies the loop region preceding α3; G249, F250, G251, and R252 are located on α3; while H284 is located on α4. Most of the residues that form hydrogen bonds with acetyl-CoA are conserved among kinetoplastids (Figure S6) and are located within motif A, which is consistent with the previous studies of acetyltransferases within the GNAT superfamily.^13,16^

### Solution structure of KKT23^125–348^ resembles the crystal structure

**Figure 3:**
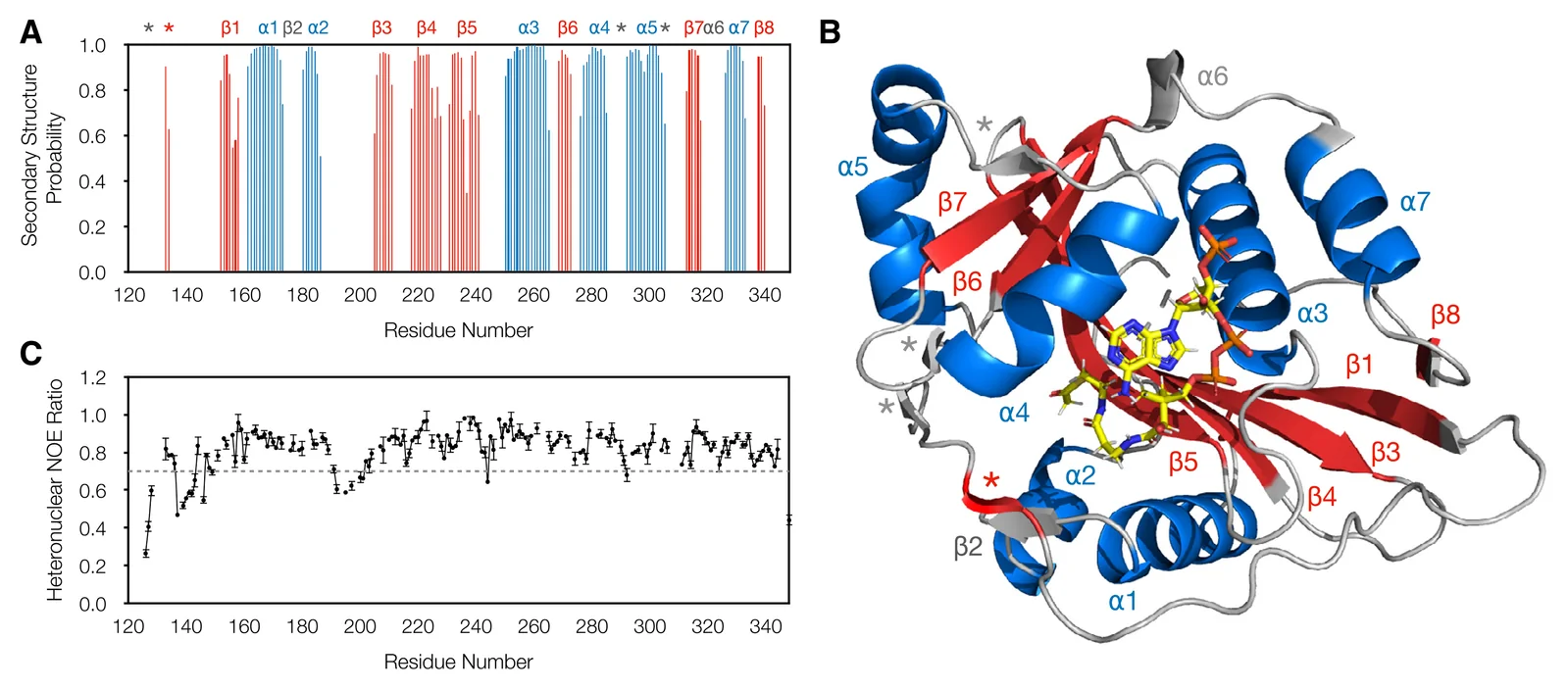
NMR analysis of KKT23^125–348^ structure and dynamics (A) The TALOS-N secondary structure analysis of KKT23^125−348^ shows a mixed α/β topology. The secondary structure elements for which the probability was lower than 0.5 are not shown in the figure. See also Data S2. (B) The secondary structure elements identified using TALOS-N (A) are highlighted in the crystal structure of KKT23^125−348^ showing a good agreement between the two methods. The β-strands and α-helices are colored in red and blue, respectively. See also Figure S4 and Data S2. (C) The {^1^H}-^15^N heteronuclear NOE ratios were measured and plotted against the sequence of KKT23^125−348^. Most of the residues display hetNOE ratios >0.7 indicating a rigid conformation of the protein backbone. The regions with higher flexibility (ratios < 0.7) include 126–128, 137–143, and 192–201. The regions between 126–128 and 137–143 are coils according to the crystal structure, whereas no electron density is visible for residues 192–200 suggesting that all three regions are part of flexible loops. The {^1^H}-^15^N hetNOE errors were estimated from 500 Monte Carlo simulations using baseline noise as a measure of peak height error.

We employed NMR spectroscopy to characterize KKT23^125−348^ in solution and to compare the structure and dynamics of KKT23^125−348^ in solution with that observed in the crystal. Using isotope-labeled KKT23^125−348^, we have obtained a detailed backbone resonance assignment in the presence of acetyl-CoA. Using the obtained chemical shifts, TALOS-N was used to predict the secondary structure elements in KKT23^125−348^ in solution,^17^ showing a mixture of α-helices and β-strands (Figure 3A; Data S2). Overall, the secondary structure elements identified by TALOS-N are in excellent agreement with those identified in the crystal structure (Figure 3B and Data S2). It is noteworthy that as in the crystal structure, the NMR analysis confirmed the lack of the predicted helix between residues 125–150. The β-strands involving residues 129–130 and 308–309 have not been predicted by TALOS-N. However, this can be explained by the lack of assignments for residues in both regions. It is likely that peaks corresponding to these residues are broadened and this may indicate conformational exchange on a slower timescale for this β-sheet in solution; future NMR studies using, for example, relaxation dispersion methods may be useful in probing slower timescale dynamics in more detail. Additionally, TALOS-N does predict the short β-strand involving residues 133–134 but shows no evidence for strand β2 (residues 175–177) (Figures 3A and S4; Data S2).

To study the fast timescale (ps to ns) dynamics of KKT23^125−348^, {^1^H}-^15^N heteronuclear nuclear Overhauser effect (hetNOE) data were collected.^18^ The measured {^1^H}-^15^N hetNOE ratios were high (>0.7) for almost all residues (Figure 3C). This means that the majority of the residues in KKT23^125−348^, even those located in most loop regions, have a rigid backbone conformation on a fast timescale. The regions between 126–128, 137–143, and 192–201 show reduced hetNOE ratios suggesting a higher flexibility compared to the rest of the protein; this is consistent with the analysis of chemical shifts by TALOS-N that shows no secondary structure for these regions in solution. Moreover, the reduced hetNOE ratios are consistent with the elevated B-factors observed for regions 126–128 and 137–143, and the absence of electron density observed for 192–200 in the crystal structure (data not shown). These results illustrate the complementary nature of the information obtained by NMR and X-ray crystallography.

### KKT23^125–348^ is a structural homolog of GCN5 acetyltransferase domain

**Figure 4:**
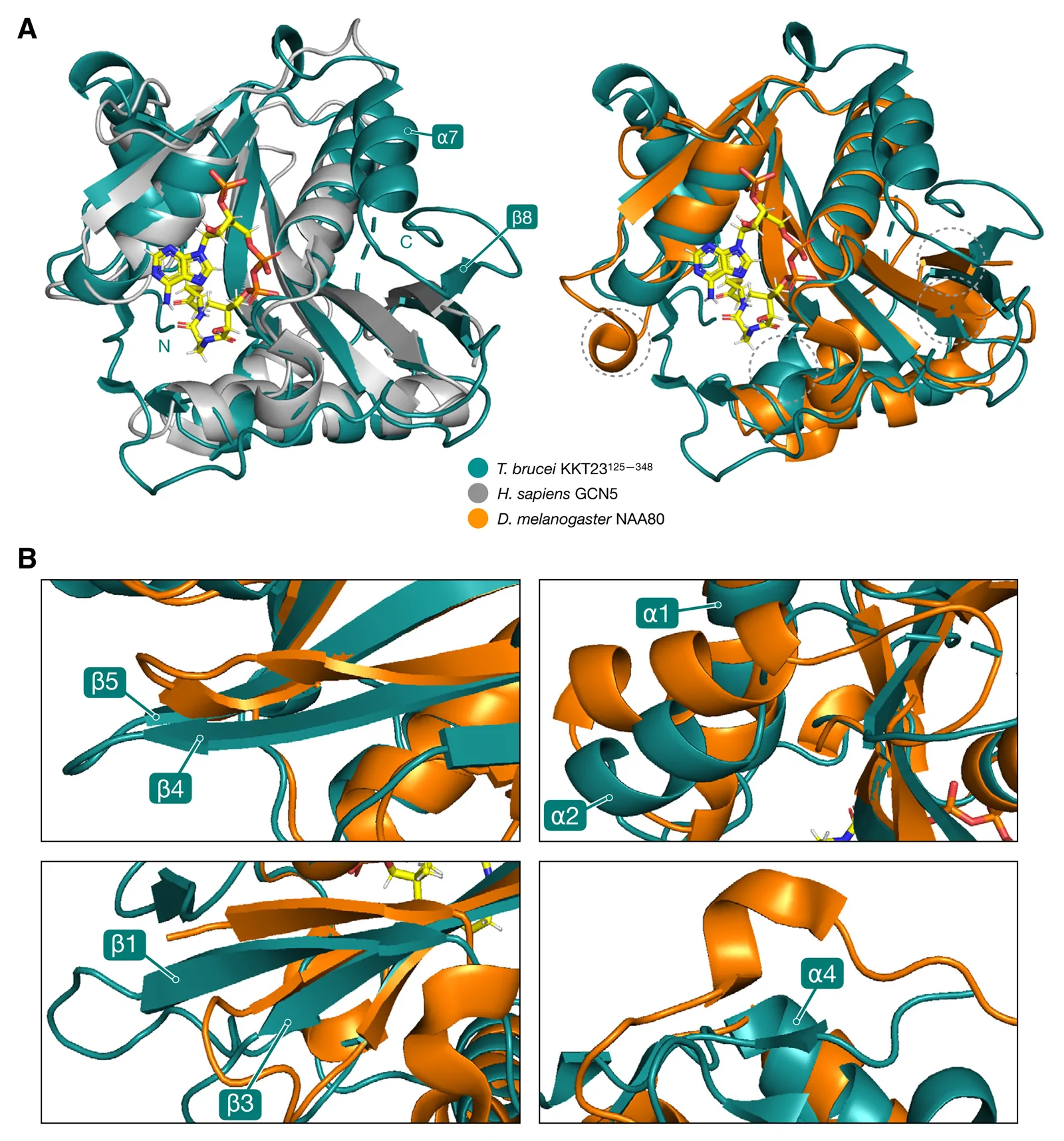
KKT23 C-terminal region is a structural homolog of GCN5 (A) Superposition of the KKT23^125−348^ (teal, PDB: 9EVQ) with *Hs*GCN5 histone acetyltransferase domain (gray, PDB: 5TRM) and *Dm*NAA80 N-terminal acetyltransferase (orange, PDB: 5WJD). The structures were overlaid using the *super* function in Pymol^14^ with an RMSD of 1.2 Å (114–114 Cα atoms) and 1.1 Å (87–87 C_α_ atoms), respectively. The lower RMSD in the overlay between KKT23^125−348^ and *Dm*NAA80 can be explained by the smaller number of atoms used for the calculation. The α7 and β8 in KKT23^125−348^ are not part of a canonical GNAT structure. The N and C represent the N- and C- termini. Regions of the structure highlighted with dotted circles represent close-up views shown in (B). (B) The images highlight the differences between KKT23 GNAT and NAA80 N-terminal acetyl-transferase domains. Most significant differences between KKT23 GNAT and NAA80 are observed within motif D, which builds the core of the GNAT domain (strands β3 and β4).

Our previous sequence analysis showed that KKT23 has a putative GNAT domain but did not allow us to determine its closest homolog.^6^ Using a structural homology search with the distance-matrix alignment (DALI) server^19^ and the crystal structure of KKT23^125−348^, we identified the human GCN5 (also known as KAT2A) histone acetyltransferase domain as the top hit (Table S1). Moreover, structural homology searches with Foldseek against the AlphaFold2-predicted structure database^20^ produced similar results (data not shown). The structural alignment between KKT23^125−348^ and human GCN5 revealed a remarkable similarity, with a root-mean-square deviation (RMSD) of 1.2 Å (114–114 Cα atoms) (Figure 4A, left panel). Notable differences between the two structures include the additional α-helix (α7) and β-strand (β8) in the crystal structure of KKT23^125−348^, which are highly conserved among kinetoplastids but are not part of the consensus GNAT domain fold (Figures 4A and S6). To highlight structural differences with other classes of the GNAT family, the crystal structure of KKT23^125−348^ was aligned with the next top hit found by the DALI search, *D. melanogaster* NAA80 N-terminal acetyltransferase (Figure 4A, right panel). The NAA80 is a member of the GNAT family that catalyzes the transfer of an acetyl group from acetyl-CoA to the N-termini of protein substrates.^21,22^ The most significant differences between KKT23 GNAT and NAA80 are observed within motif D, which builds the core of the GNAT domain (strands β3 and β4) (Figure 4B). Taken together, our structural characterization reveals that KKT23 has an acetyltransferase fold with the closest structural similarity to GCN5 histone acetyltransferases.

### KKT23 can acetylate *T. brucei* nucleosomes *in vitro*

**Figure 5:**
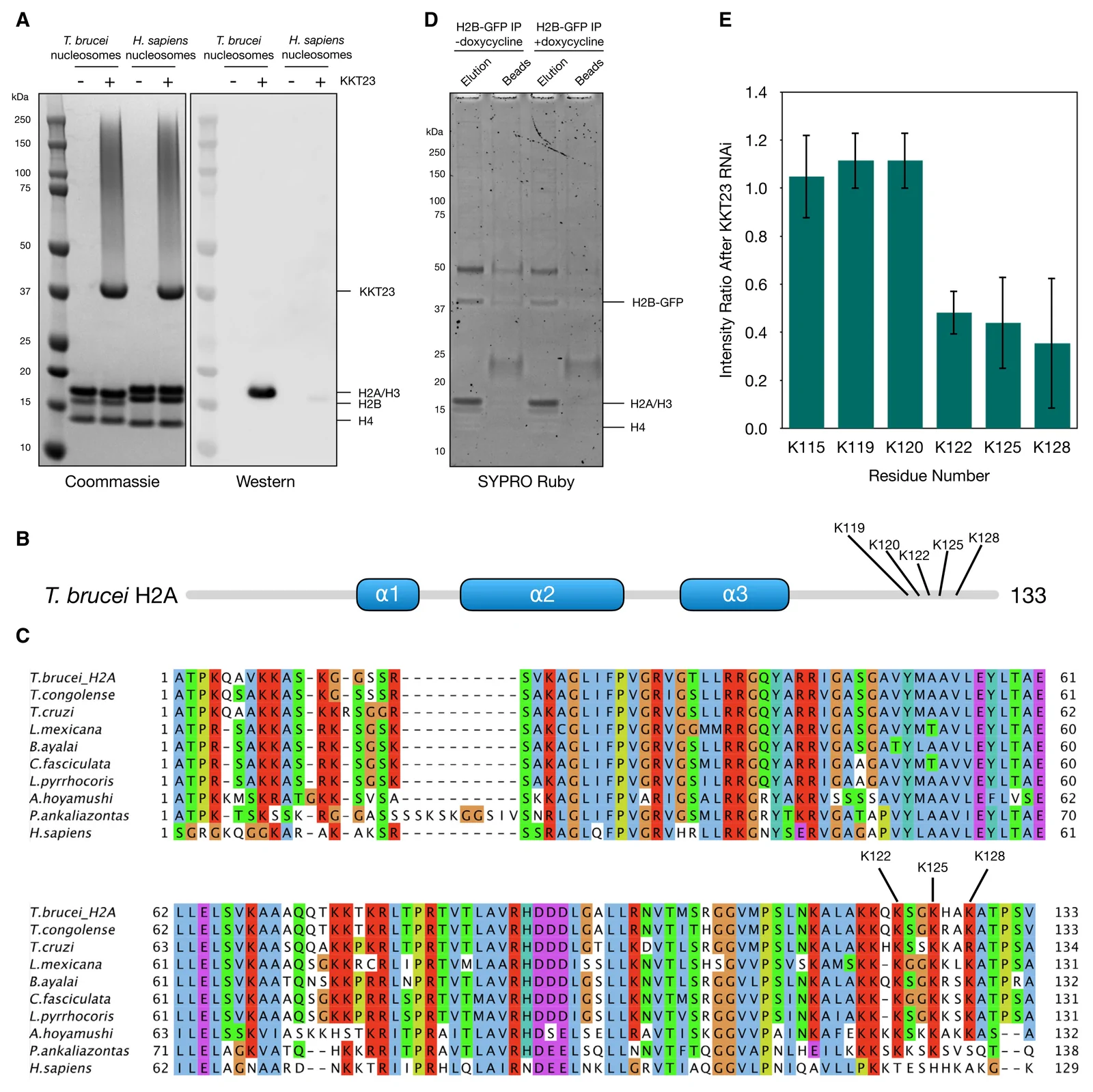
KKT23 can acetylate histone H2A (A) The SDS-PAGE gel (left panel) showing histone acetyltransferase assay on reconstituted *T. brucei* and *H. sapiens* nucleosomes using KKT23 acetyl-transferase. Histones H2A and H3 from *T. brucei* and *H. sapiens* H2A and H2B could not be resolved because of their similarity in molecular weight. Panel on the right shows Western blotting analysis using the pan-acetyl-lysine antibody. Clear signal can be observed near the molecular weight of *T. brucei* H2A/H3, while no signal is observed with the rest of the *T. brucei* histones or with *H. sapiens* nucleosomes, indicating that KKT23 is specific toward *T. brucei* nucleosomes. (B) Schematic of *T. brucei* H2A sequence. The C-terminal lysines robustly acetylated by KKT23 in (A) are annotated. *In vitro* acetylation experiments were performed three times (*n* = 3). See also Table S2. (C) Sequence alignment of histone H2A sequences from kinetoplastid species and human. Acetylation sites that exhibited reduced levels following KKT23 RNAi (E) are highlighted. (D) SDS-PAGE gel stained with SYPRO Ruby showing immunoprecipitation of H2B-GFP from *T. brucei* cells subjected to KKT23 RNAi (+doxycycline). Control experiment was done using the same cell line but without inducing RNAi (-doxycycline). Histone H2B fused to GFP runs ~37 kDa and co-purifies with core histones. Tubulin contamination can be observed at ~50 kDa. Immunoprecipitation experiments were performed at least three times. Cell line, BAP2596. See also Figure S7. (E) Mass spectrometry analysis of the histone H2A acetylated sites identified in the procyclic form of *T. brucei* cells, 24 h after induction of KKT23 RNAi. Intensities of the acetylated peptides were normalized against intensities of total H2A in control and RNAi samples individually. Two-tailed unpaired t tests show that the reduction of acetylation levels on three sites is statistically significant (*p* < 0.005 for K122, *p* < 0.05 for K125 and K128). The other sites (K115, K119, and K120) did not show significant changes (*p* > 0.05). None of other histone acetylation sites that were detected in all experiments showed significant reduction (Table S3). Immunoprecipitation and mass spectrometry were performed at least three times. Error bars represent the standard deviation.

GCN5 is found in numerous complexes in other well studied eukaryotes and is known to acetylate histone N-terminal tails.^23–26^ The structural similarity between the KKT23 GNAT domain and the GCN5 histone acetyltransferase domain raised the possibility that KKT23 may acetylate trypanosome histones. To test this hypothesis, we performed an *in vitro* histone acetyltransferase assay. Briefly, recombinant KKT23 was incubated with acetyl-CoA and nucleosomes reconstituted using either *T. brucei* or *Homo sapiens* histones.^27^ Then, the reactions were analyzed by immunoblots using anti-acetyl-lysine antibody. The acetylation signal was detected around the position of *T. brucei* H2A and H3 histones in the gel, but not when *H. sapiens* nucleosomes were used, suggesting that KKT23 is specific toward *T. brucei* nucleosomes (Figure 5A). To reveal the identity of the acetylated histone(s) and map the acetylated sites, mass spectrometry was employed. Gel bands corresponding to individual histones (H2A/H3, H2B, and H4) were excised, digested with trypsin, and analyzed by mass spectrometry. We found that lysine residues on the C-terminal tail of H2A were robustly acetylated (K119, K120, K122, K125, and K128), while acetylation on H2B, H3, and H4 or other sites in H2A was less significant (Table S2; Figure 5B). KKT23 therefore has different substrate specificity compared to GCN5 acetyltransferases in other eukaryotes that are known to preferentially acetylate histones H3 and H4 but not H2A.^23–26^

### KKT23 depletion leads to reduction of H2A C-terminal acetylation in *T. brucei*

Previous studies established that the C-terminal tail of histone H2A is hyperacetylated in trypanosomatids, a feature that is not found in human H2A,^28–32^ but the enzyme responsible for this acetylation was unknown.^33^ To test a possibility that KKT23 contributes to the H2A hyperacetylation, we analyzed histone acetylation levels by immunoprecipitating histone H2B-GFP from *T. brucei* cells. Using fluorescence microscopy, we confirmed that H2B-GFP was distributed in the nuclei (Figure S7) and that other core histones co-purified with it (Figure 5D). We previously showed that the KKT23 protein level was reduced 2 days after RNAi induction (in ~60% of cells), while the growth retardation was evident only after 4 days.^9^ The acetylation levels of K122, K125, and K128 were reduced by around half after depleting KKT23 with RNAi for 2 days, while those of K115, K119, and K120 did not change (Figures 5E and 5C; Table S3). These results show that the hyperacetylation of the histone H2A C-terminal tail partly depends on KKT23 *in vivo*.

### KKT23 forms a complex with KKT22 and KKT3

**Figure 6:**
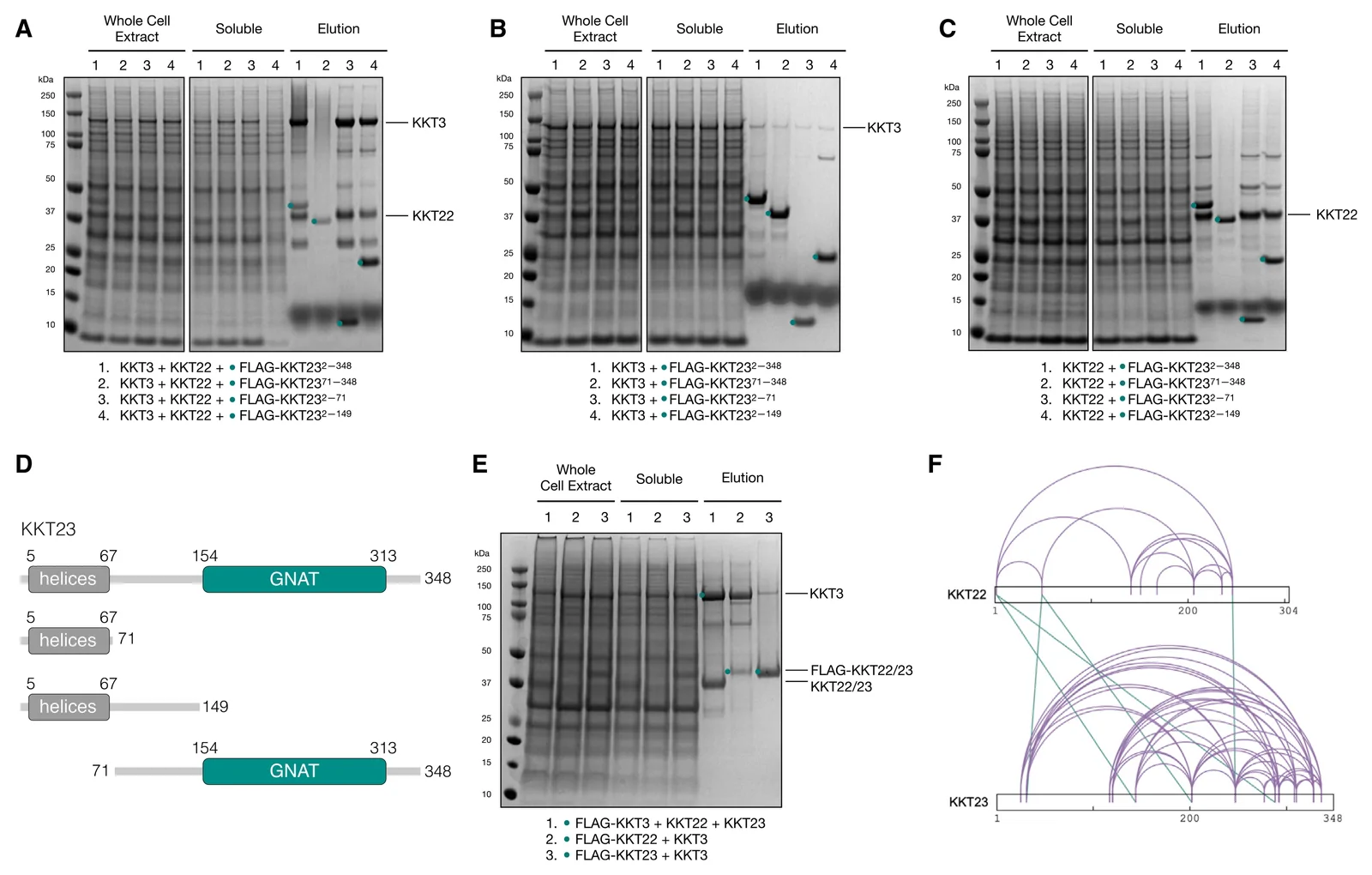
KKT23 forms a complex with KKT3 and KKT22 *in vitro* (A–C) SDS-PAGE showing immunoprecipitation of FLAG-KKT23 fragments with KKT3 and KKT22 (A), KKT3 (B), and KKT22 (C). The migration of FLAG-tagged fragments is labeled with teal dots. FLAG-KKT23 pulls down KKT3 and KKT22 when three proteins are co-expressed in Sf9 insect cells. However, KKT23 is not able to pull down KKT3 in the absence of KKT22. KKT23 N-terminal fragment is sufficient to reconstitute the complex composed on three proteins. Each immunoprecipitation experiment was performed once. (D) Cartoon schematic depicting boundaries of KKT23 fragments used in (A–C). (E) SDS-PAGE showing reconstitution of complexes using full-length KKT3, KKT22, and KKT23 expressed in Sf9 cells. FLAG-KKT3 can pull down both KKT22 and KKT23 (lane 1). Bands corresponding to untagged KKT22 and KKT23 overlap in the gel. The presence of both proteins in the sample was confirmed by mass spectrometry (data not shown). In contrast to FLAG-KKT22 (lane 2), FLAG-KKT23 cannot pull down KKT3 efficiently (lane 3). Immunoprecipitation of full-length proteins was performed twice (*n* = 2) and identity of the proteins were verified using mass spectrometry. See also Figure S8. (F) Crosslinking mass spectrometry analysis of a complex composed of KKT3^Δ594-729^, KKT22 and FLAG-KKT23, crosslinked with 220 μM BS^3^. Due to DNA-contamination of the complex containing the full-length KKT3, deletion mutant of KKT3 was used, where its DNA-binding centromere-localization domain was removed. The panel illustrates crosslinks detected between KKT22 and KKT23 (green lines). No crosslinks were detected between KKT3 and KKT22 or KKT23. A complete list of identified crosslinks is shown in Table S4.

Our previous immunoprecipitation experiments suggest that KKT23, KKT22, and KKT3 are closely associated.^6^ Furthermore, RNAi-mediated knockdown of KKT3 causes localization defects for KKT22 and KKT23, while depletion of KKT22 or KKT23 did not affect KKT3 localization.^9^ To test whether KKT23, KKT22, and KKT3 directly interact, these proteins were co-expressed in insect cells (Sf9) and pulled down using FLAG-KKT23 (Figure 6A). Indeed, FLAG-KKT23 co-purified with KKT22 and KKT3 confirming a direct interaction between these proteins (Figure 6A). To dissect the hierarchy of these interactions, we repeated the experiment by omitting either KKT3 or KKT22 expression. KKT3 did not significantly co-purify with FLAG-KKT23 in the absence of KKT22 expression (Figure 6B), while KKT22 co-purified with FLAG-KKT23 even when KKT3 was absent (Figure 6C). These results suggest that KKT23 directly interacts with KKT22 and that KKT22 is required to reconstitute the complex composed of KKT3, KKT22. and KKT23 in this assay. Consistent with this possibility, KKT3 co-purified with FLAG-KKT22, but not with FLAG-KKT23 (Figure 6E). We next conducted crosslinking experiments on the purified KKT3-KKT22-KKT23 complex using BS^3^ (bis(sulfosuc-cinimidyl)suberate) and analyzed the samples by mass spectrometry (Figure 6F; Table S4). The results revealed multiple crosslinks between KKT22 and KKT23, but none were detected between KKT3 and KKT22 or KKT23. It is worth noting that numerous crosslinks were detected between the KKT23 N-terminal region and the acetyltransferase domain, implying a potential regulation of the KKT23 GNAT domain by the N-terminal region.

### N-terminal fragment of KKT23 interacts with KKT22

**Figure 7:**
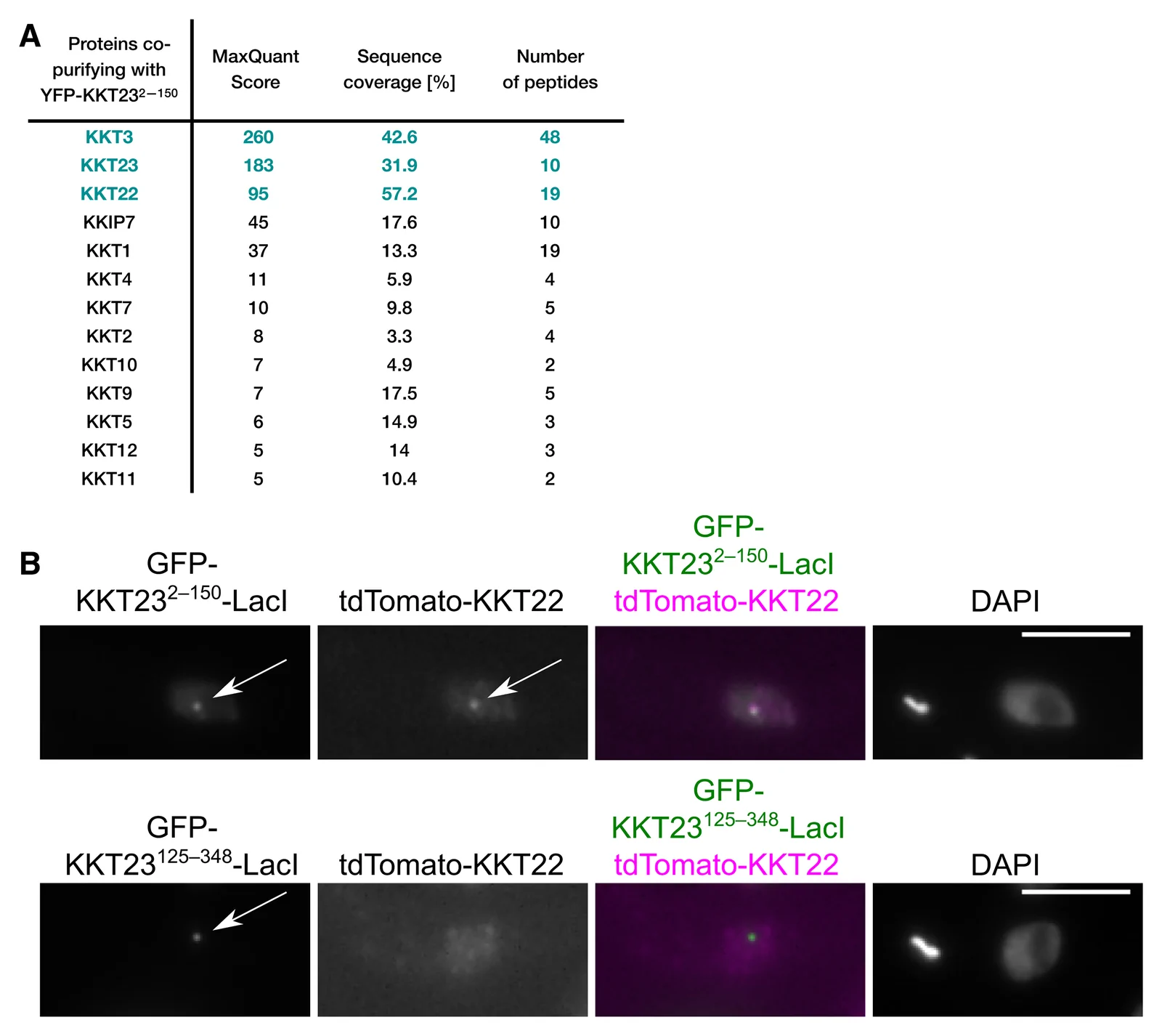
KKT23^2–150^ is sufficient to interact with kinetochore proteins (A) Mass spectrometry analysis of YFP-KKT23^2−150^ immunoprecipitated using anti-GFP antibodies from *T. brucei* cells. The N-terminal fragment of KKT23 co-purifies with a number of kinetochore proteins with KKT3 and KKT22 being the top hits. See Table S5 for all proteins identified by mass spectrometry. The immunoprecipitation experiment was performed once. Cell line, BAP2296. (B) The N-terminal fragment of KKT23 (KKT23^2−150^,top panel) but not the C-terminal fragment (KKT23^125−348^, lower panel) is sufficient to recruit KKT22 to a non-centromeric locus in *T. brucei* cells (5 cells each). The expression of GFP-fused proteins was induced with 10 ng/mL doxycycline for 24 h. Cell lines, BAP2547 and BAP2546. Scale bar, 5 μm.

To determine the region of KKT23 that interacts with KKT22, the pull downs were repeated with FLAG-tagged fragments of KKT23 (Figure 6D). Reconstitution of the three-protein complex could only be observed with the N-terminal fragments of KKT23 (KKT23^2−70^ and KKT23^2−149^) but not with its C-terminal fragment (KKT23^71−348^) (Figure 6A). Like full-length KKT23, the N-terminal fragment of KKT23 co-purified with KKT22 even in the absence of KKT3 (Figure 6C). Based on the observation that the N-terminal region of KKT23 is sufficient to interact with KKT22 in this assay, KKT23^2−150^ was ectopically expressed in *T. brucei* cells and immunoprecipitated to analyze the co-purifying proteins. Indeed, mass spectrometry of the YFP-KKT23^2−150^ immunoprecipitation sample revealed co-purification of many kinetochore proteins, with KKT3 and KKT22 being the top hits (Figure 7A). We next employed a LacO-LacI system^34^ to tether the KKT23^2−150^-LacI protein to a non-centromeric locus and found recruitment of KKT22 (Figure 7B). In contrast, no recruitment was observed when KKT23^125−348^-LacI was used (Figure 7B). These results in combination with the immunoprecipitation experiments show the importance of the KKT23 N-terminal region for the interaction with KKT22.

### Crystal structure of KKT23^2–70^

**Figure 8:**
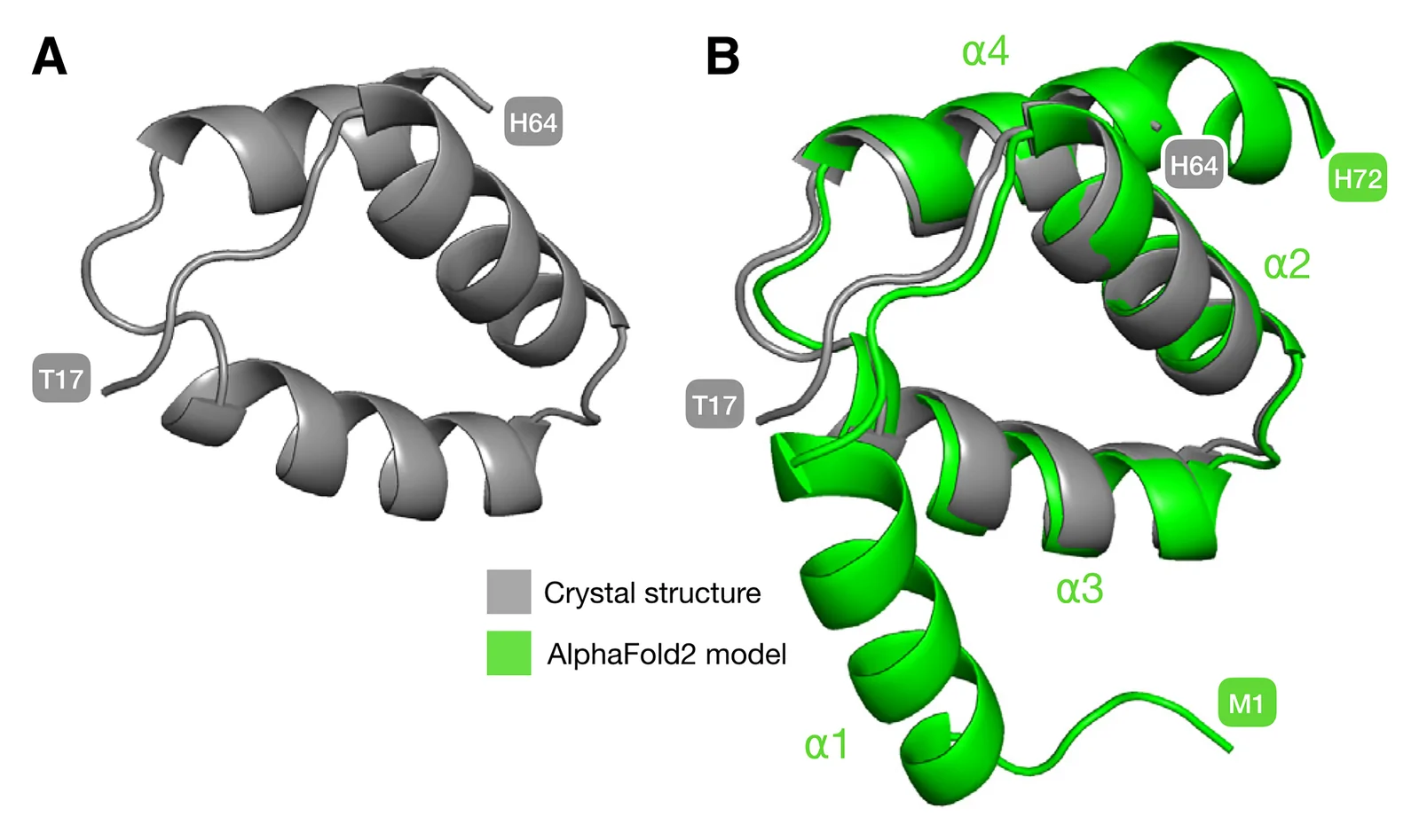
Crystal structure of KKT23^2–70^ reveals helical fold within its N-terminal region (A) Ribbon representation of the KKT23^2−70^ structure determined by X-ray crystallography. The model starts at T17 and ends at H64. Residues between 2–16 and 65–70 were not modeled as no electron density was observed for these regions. (B) Superposition of the KKT23^2−70^ (gray) with the AlphaFold2 model of KKT23^2−72^ (green). The structures were overlaid using an *align* function in Pymol^14^ with an RMSD of 0.8 Å (44–44 Cα atoms). AlphaFold2 predicted an additional N-terminal helix between residues 5–17. No electron density was observed in the crystal structure for this region.

Given the importance of the KKT23 N-terminal region, we next aimed to determine its structure using X-ray crystallography. Purification and crystallization screening of KKT23^2−70^ yielded well diffracting crystals that allowed us to solve the structure at 2.7 Å resolution (Figure 8A; Table 1). The structure comprises three short helices between residues 22–33, 36–49, and 54–62. Due to the lack of electron density in the structure, residues between 2–14 and 65–70 were not modeled. Consequently, we were unable to confirm the secondary structure prediction, which placed an additional helix between residues 5–17 (Figure S1A). To address this issue, we used AlphaFold2 to predict the structure of KKT23^2−72^ (Figure 8B), which indeed revealed an additional helix between residues 5–17 and a longer helix at the C-terminus. Notably, the predicted structure closely matched our crystal structure with an RMSD of 0.8 Å (44–44 Cα atoms). The DALI search of the crystal structure or the AlphaFold2 model of the KKT23 N-terminal domain did not reveal any obvious structural homolog (data not shown). We next used AlphaFold-Multimer and found that the KKT23 N-terminal region is predicted to interact with KKT22 (Figure S8). The helices α1–α3 from KKT23 are predicted to interact with α2 and α6 of KKT22 in multiple models with high pLDTT scores through hydrophobic and electrostatic interactions.

### The N-terminal helical domain and the C-terminal GNAT domain are linked by a stretch of unstructured residues

We have determined the structures of KKT23^2−70^ and KKT23^125−348^. However, the region between these two domains, encompassing residues 72–126, is predicted to be disordered (Figure S1B). To gain insight into this region’s structural details, we expressed and purified ^15^N-labeled full-length KKT23. KKT23^2−348^ gives a well-resolved ^1^H-^15^N BEST TROSY spectrum. Many of the observed peaks align well with the peaks in the ^1^H-^15^N BEST TROSY spectrum of KKT23^125−348^ (Figure S9); therefore, these peaks were assigned to the GNAT domain. Most of the remaining peaks displayed the higher intensity and narrow dispersion in the ^1^H dimension (Figure S10A), which are often observed for unstructured protein regions. To probe the dynamics of the residues giving rise to these intense peaks, the {^1^H}-^15^N heteronuclear NOE was measured (Figure S10B).^18^ The analysis revealed hetNOE values below 0.4 for the 39 strongest peaks, indicating that they correspond to unstructured regions of KKT23, most likely the region between residues 72–126. In fact, some of these peaks were assigned to residues in the region of A91 to E100 (Figure S10A). In conclusion, our data show that the structure of KKT23 consists of an N-terminal helical region, a stretch of unstructured residues, and the GNAT domain at the protein C-terminus.

## Discussion

KKT23 is a unique kinetochore protein that contains a GNAT domain. The fact that KKT23 is conserved in all sequenced kinet-oplastids implies that it may play fundamental roles in kineto-plastid kinetochores.^6^ Although our previous study showed that KKT23 is essential for cell growth, its structure and function were unknown. Using a combination of X-ray crystallography and NMR spectroscopy, we characterized the structure of KKT23^125−348^, which adopts an acetyltransferase fold, with GCN5 histone acetyltransferase being its closest structural homolog. NMR, which allows the study of protein structure in solution, free from the constraints of a crystal lattice, enabled us to probe the dynamics of KKT23 GNAT domain at single-residue resolution. This revealed regions of higher flexibility, consistent with the B-factors observed in the crystal structure. We were unable to crystallize the full-length KKT23, which, in addition to the GNAT domain, includes the N-terminal helical domain and an unstructured region. In the BEST TROSY spectrum of the fulllength KKT23, we were able to observe peaks corresponding to the unstructured region and investigate its dynamics, confirming that it is disordered; this is something that X-ray crystallog-raphy could not achieve.

In line with this discovery that KKT23^125−348^ has an acetyltransferase fold, with the GCN5 histone acetyltransferase being its closest structural homolog, we demonstrate that KKT23 acetylates the C-terminal tail of histone H2A from *T. brucei*, while no activity was observed toward human histones. The substrate specificity of KKT23 can be attributed to several factors. Notably, the acetylation sites identified in the C-terminal tail of *T. brucei* H2A (K122, K125, and K128) are separated by two-residue gaps, a feature absent in the human H2A C-terminal tail. Furthermore, there is a slight difference in the overall isoelectric point (pI) of the *T. brucei* H2A C-terminal tail (10.2) compared to its human counterpart (10.7). Our recent cryoelectron microscopy (cryo-EM) structure of *T. brucei* nucleosomes^27^ revealed that the C-terminal tail of H2A is more flexible than that of human H2A. This increased flexibility, likely due to reduced hydrophobic interactions between the *T. brucei* H2A C-terminal tail and the H3 αN and α2 helices, may contribute to different substrate specificity. Additionally, substrate specificity may be regulated in cells by interactions with other kinetochore proteins or post-translational modifications (PTMs) on histones.

It has been known that the C-terminal tail of histone H2A is hyperacetylated in trypanosomes (K115, K119, K120, K122, K125, and K128).^28–32^ A recent study estimated that ~20% of K115, K119, and K120 and 5%–10% of K122, K125, and K128 is acetylated in proliferating *T. brucei* bloodstream form cells.^32^ The enzyme responsible for these acetylation events remained unknown. Our study provides a partial answer for this long-standing question by showing that KKT23 can acetylate these sites *in vitro* and that its knockdown leads to a significant decrease in acetylation levels of K122, K125, and K128 in trypanosome cells. However, it is important to note that the acetylation levels of K115, K119, and K120 remained unchanged after KKT23 depletion, suggesting that other enzymes are responsible for the acetylation of these C-terminal lysines. Besides KKT23, there are at least six other putative histone acetyltransferases in *T. brucei*.^35^ It will be important to identify which acetyltransferase(s) are responsible for the H2A C-terminal acetylation in the future. A previous study performed chromatin immunoprecipitation sequencing (ChIP-seq) using antibodies against the acetylated C-terminal tail (raised against peptides with K115acK120acK122ac or K120ac-K122acK125ac) and found enrichment at Pol II transcription start regions.^36^ Based on the finding that acetylation levels of only K122, K125, and K128 were reduced after KKT23 depletion, it will be important to examine whether acetylation of these specific sites is enriched at the centromeric region. It will also be important to reveal the functional significance of histone H2A acetylation by KKT23. Our qualitative ChIP-seq analysis of KKT3 after depleting KKT23 for 4 days failed to find any change in the position of kinetochore assembly sites (Figure S11). We speculate that KKT23-dependent acetylation of histone H2A could alter chromatin structure directly; acetylation may neutralize lysine’s positive charge and thus destabilize the interaction between the DNA and histones.^37,38^ The C-terminal lysine residues are not ordered in known nucleosome structures, but are likely adjacent to linker DNA based on the presence of the H2A C-terminal tail near the entry-exit site of DNA. This could subsequently lead to an enhanced DNA accessibility and transcription.^39^ Indeed, transcription has been detected at centromeres in various eukaryotes and is thought to play important roles in ensuring proper kinetochore functions.^40^ In *T. brucei*, small non-coding RNA has been detected for the centromeric repeat sequence of several chromosomes.^41^ Therefore, KKT23’s acetyltransferase activity may play an important role in ensuring proper kinetochore function by regulating centromeric chromatin. The structural and biochemical insights generated from our analysis of KKT23, a unique kinetochore protein, could open up potential avenues for the rational design of drugs aimed at combating kinetoplastid-associated diseases in the future.

## Star⋆Methods

Detailed methods are provided in the online version of this paper and include the following:

- KEY RESOURCES TABLE
- EXPERIMENTAL MODEL AND STUDY PARTICIPANT DETAILS
  ∘ Bacterial culture
  ∘ Cell culture
- METHOD DETAILS
  ∘ Plasmids
  ∘ Protein expression and purification
  ∘ Expression of isotopically labeled KKT23 fragments
  ∘ Expression and purification from insect cells
  ∘ Size Exclusion Chromatography with Multi-Angle Light Scattering
  ∘ Analytical Ultracentrifugation
  ∘ Crystallization and structure determination
  ∘ NMR spectroscopy and analysis of NMR data
  ∘ Reconstitution of *T. brucei* and *H. sapiens* nucleosome core particles
  ∘ *In vitro* histone acetyltransferase assays on reconstituted nucleosomes
  ∘ Trypanosome cells
  ∘ Microscopy
  ∘ Immunoprecipitation of histones and KKT23^2−150^ from trypanosomes
  ∘ Mass spectrometry: In-solution trypsin digest for immunoprecipitates of H2B-GFP and GFP-KKT23^2−150^ purified from trypanosomes
  ∘ Mass spectrometry: In-gel digestion for nucleosomes acetylated using recombinant KKT23
  ∘ Chemical crosslinking mass spectrometry (XL-MS) of KKT23 complex
  ∘ Multiple sequence alignment
  ∘ *In silico* structure and interaction predictions
- QUANTIFICATION AND STATISTICAL ANALYSIS

## Star⋆Methods

### Key Resources Table

**Table:** 

REAGENT or RESOURCE	SOURCE	IDENTIFIER
Antibodies		
Acetylated-Lysine (Ac-K2-100) MultiMab™ rabbit mAb mix	Cell Signaling Technology	9814S; RRID: AB_10544700
Rabbit IgG HRP-linked whole antibody	VWR	NA934-1ML; RRID: AB2722659
Mouse monoclonal anti-GFP	Roche	11814460001; RRID: AB_390913
Rabbit polyclonal anti-GFP	Thermo Fisher	A11122; RRID: AB_221569
Bacterial and virus strains		
*Escherichia coli:* BL21(DE3)	Novagen	69450
Chemicals, peptides, and recombinant proteins		
2-chloroacetamide	Sigma-Aldrich	C0267
3xFLAG peptide	Sigma-Aldrich	F4799
Acetyl coenzyme A trisodium salt (acetyl-CoA)	Sigma-Aldrich	A2056
Ammonium chloride (15N, 99%)	Goss Scientific	NLM-467
Anti-FLAG M2 affinity gel	Sigma-Aldrich	A2220
Benzonase nuclease	Sigma-Aldrich	E1014
BS^3^_-d0_	Thermo Fisher	SK256507
Cellfectin II	Invitrogen	10362-100
Coenzyme A sodium salt hydrate (CoA)	Sigma-Aldrich	C3144
D-Glucose (13C6, 99%)	Goss Scientific	CLM-1396
Deuterium oxide	Sigma-Aldrich	151882
Dimethyl pimelimidate dihydrochloride (DMP)	Sigma-Aldrich	D8388
Dithiothreitol (DTT)	Fisher	10578170
Doxycycline	Sigma-Aldrich	D9891
E–64	Peptide Institute Inc.	4096
EDTA	Sigma-Aldrich	E4884
EGTA	Sigma-Aldrich	E3889
Formaldehyde (16%)	ThermoFisher	28906
Glycerol	Sigma-Aldrich	G5516
Heat-inactivated metal calf serum (FBS)	Scientific Laboratory Supplies	F4135
HEPES	Sigma-Aldrich	H3375
HiLoad 16/600 Superdex 75 pg	Cytiva	28-9893-33
Igepal CA-630 (NP-40)	Sigma-Aldrich	I8896
Imidazole	Sigma-Aldrich	56750
IPTG	Sigma-Aldrich	I6758
Kanamycin sulfate	Cambridge Bioscience Ltd	15321-25 g-CAY
KCl	Sigma-Aldrich	P9541
L-(+)-Selenomethionine, anagrade	Molecular Dimensions	S2000-1-GM
Leupeptin	EMD Milipore Corp.	3158107
MgCl_2_	Sigma-Aldrich	M8266
MgSO_4_	Sigma-Aldrich	M7506
Microcystin-LR	Enzo Life Sciences (UK)Ltd	ALX-350-012-C500
Na-beta-glycerophosphate	Sigma-Aldrich	50020
Na_2_HPO_4_	Sigma-Aldrich	S0876
Na_3_VO_4_	Sigma-Aldrich	S6508
NaCl	Sigma-Aldrich	S9888
NaF	Sigma-Aldrich	S7920
NaH_2_PO_4_	Sigma-Aldrich	S0751
Pepstatin A	EMD Milipore Corp.	516481
PIPES	Sigma-Aldrich	P6757
PMSF	Sigma-Aldrich	P7626
Protein A magnetic beads (Dynal)	Life Technologies	10002D
Protein G Dynabeads	Life Technologies	10004D
Resource Q 6 mL anion exchange chromatography column	Cytiva	17-1179-01
Resource S 6 mL cation exchange chromatography column	Cytiva	17-1180-01
SDM-79 media for trypanosome culture	Invitrogen	N/A
Sequencing Grade Modified Trypsin	Promega	V5111
Sf-900 II SFM media	Invitrogen	10902104
SimplyBlue Safe Stain	Invitrogen	46-5034
Sodium butyrate	Alfa Aesar	A11079.22
Sodium dodecyl sulfate (SDS)	Sigma-Aldrich	75746
Sodium pyrophosphate	Sigma-Aldrich	221368
Superdex 75 10/300	Cytiva	17-5174-01
TALON metal affinity resin	Takara	635503
TCEP	Sigma-Aldrich	C4706
Tris base	Sigma-Aldrich	252859
SuperSignal Western pico chemiluminescent substrate	ThermoFisher	34577
Critical commercial assays		
MIDASplus HT-96 crystallization screen	Molecular Dimensions	MD1-107
ProPlex HT-96 crystallization screen	Molecular Dimensions	MD1-42
SelenoMethionine medium base plus nutrient mix	Molecular Dimensions	MD12-501
Deposited data		
ChIP-seq of KKT3-YFP before and after induction of KKT23 RNAi, raw data	This study	ENA: PRJEB76406
Crosslinking mass spectrometry, raw data	This study	PRIDE: PXD052923
*In vitro* acetyltransferase assay: H2A, H3 experiment 1, control, raw data	This study	PRIDE: PXD052922
*In vitro* acetyltransferase assay: H2A, H3 experiment 1, KKT23 RNAi, raw data	This study	PRIDE: PXD052922
*In vitro* acetyltransferase assay: H2A, H3 experiment 2, control raw, data	This study	PRIDE: PXD052922
*In vitro* acetyltransferase assay: H2A, H3 experiment 2, KKT23 RNAi, raw data	This study	PRIDE: PXD052922
*In vitro* acetyltransferase assay: H2A, H3 experiment 3, control, raw data	This study	PRIDE: PXD052922
*In vitro* acetyltransferase assay: H2A, H3 experiment 3, KKT23 RNAi, raw data	This study	PRIDE: PXD052922
*In vitro* acetyltransferase assay: H2B experiment 1, control, raw data	This study	PRIDE: PXD052922
*In vitro* acetyltransferase assay: H2B experiment 1, KKT23, RNAi raw data	This study	PRIDE: PXD052922
*In vitro* acetyltransferase assay: H2B experiment 2, control, raw data	This study	PRIDE: PXD052922
*In vitro* acetyltransferase assay: H2B experiment 2, KKT23 RNAi, raw data	This study	PRIDE: PXD052922
*In vitro* acetyltransferase assay: H2B experiment 3, control, raw data	This study	PRIDE: PXD052922
*In vitro* acetyltransferase assay: H2B experiment 3, KKT23 RNAi, raw data	This study	PRIDE: PXD052922
*In vitro* acetyltransferase assay: H4 experiment 1, control, raw data	This study	PRIDE: PXD052922
*In vitro* acetyltransferase assay: H4 experiment 1, KKT23, RNAi raw data	This study	PRIDE: PXD052922
*In vitro* acetyltransferase assay: H4 experiment 2, control, raw data	This study	PRIDE: PXD052922
*In vitro* acetyltransferase assay: H4 experiment 2, KKT23 RNAi, raw data	This study	PRIDE: PXD052922
*In vitro* acetyltransferase assay: H4 experiment 3, control, raw data	This study	PRIDE: PXD052922
*In vitro* acetyltransferase assay: H4 experiment 3, KKT23 RNAi, raw data	This study	PRIDE: PXD052922
IP-MS: GFP-KKT23^2–150^ (KKT23N), raw data	This study	PRIDE: PXD052922
IP-MS: H2B-GFP experiment 1, control, raw data	This study	PRIDE: PXD052922
IP-MS: H2B-GFP experiment 1, KKT23 RNAi, raw data	This study	PRIDE: PXD052922
IP-MS: H2B-GFP experiment 2, control, raw data	This study	PRIDE: PXD052922
IP-MS: H2B-GFP experiment 2, KKT23 RNAi, raw data	This study	PRIDE: PXD052922
IP-MS: H2B-GFP experiment 3, control, raw data	This study	PRIDE: PXD052922
IP-MS: H2B-GFP experiment 3, KKT23 RNAi, raw data	This study	PRIDE: PXD052922
KKT23^125–348^ + acetyl-CoA chemical shifts	This study	BMRB: 52461
*Trypanosoma brucei* KKT23^2–70^ crystal structure	This study	PDB: 9EVR
*Trypanosoma brucei* native KKT23^125–348^ crystal structure	This study	PDB: 9EVQ
*Trypanosoma brucei* selenomethionine KKT23^125–348^ crystal structure	This study	PDB: 9F5Q
Experimental models: cell lines		
*T. brucei:* H2B-GFP, BAP249	This study	N/A
*T. brucei:* H2B-GFP, KKT23 RNAi, BAP2596	This study	N/A
*T. brucei:* tdTomato-KKT1, BAP171	This study	N/A
*T. brucei:* tdTomato-KKT1, GFP-NLS-KKT23^2–150^ (KKT23N), BAP2296	This study	N/A
*T. brucei:* LacO at rDNA, BAP347	(Ishii and Akiyoshi)^42^	N/A
*T. brucei:* LacO, TY-tdTomato-KKT22, BAP2158	This study	N/A
*T. brucei*: LacO, TY-tdTomato-KKT22, GFP-NLS-KKT23^2–150^ (KKT23N)-LacI, BAP2547	This study	N/A
*T. brucei*: LacO, TY-tdTomato-KKT22, GFP-NLS-KKT23^121–348^ (KKT23C)-LacI, BAP2546	This study	N/A
*T. brucei:* KKT3-YFP, BAP1639	(Marcianò et al.)^9^	N/A
*T. brucei:* KKT3-YFP, KKT23 RNAi, BAP2021	(Marcianò et al.)^9^	N/A
Experimental models: organisms/strains		
*Spodoptera frugiperda:* Sf9	Thermo Fisher	12659017
*Trypanosoma brucei brucei* TREU 927/4 procyclic cells expressing T7 RNA polymerase and the tetracycline repressor to allow inducible expression	(Poon et al.)^43^	SmOxP9
Oligonucleotides		
Oligonucleotides used in this study are listed in Table S6	This study	N/A
Recombinant DNA		
Sequences of synthetic DNA used in this study are listed in Table S6	This study	N/A
Plasmid: pRSFDuet-1	Novagen	71341
Plasmid: pNIC28-Bsa4	(Gileadi et al.)^44^	N/A
Plasmid: pACEBac1	Geneva Biotech	N/A
Plasmid: pACEBac2	Geneva Biotech	N/A
Plasmid: Endogenous YFP tagging vector, hygromycin, pBA1	(Kelly et al.)^45^	pEnT5-Y
Plasmid: TY-YFP-KKT1, endogenous locus, pBA162	(Marcianò et al.)^9^	N/A
Plasmid: Inducible expression vector, pBA310	(Nerusheva and Akiyoshi)^5^	N/A
Plasmid: Inducible GFP-NLS-KKT23^2–150^ (KKT23N) expression, pBA2535	This study	N/A
Plasmid: H2B-GFP, tubulin locus, pBA236	This study	N/A
Plasmid: KKT23 hairpin RNAi, pBA2021	(Marcianò et al.)^9^	N/A
Plasmid: 256LacO, integrate at rDNA, pBA152	(Landeira and Navarro)^34^	pMig96
Plasmid: Endogenous tdTomato tagging vector, hygromycin, pBA892	(Ishii and Akiyoshi)^42^	N/A
Plasmid: TY-YFP-KKT22, endogenous locus, pBA1714	This study	N/A
Plasmid: TY-tdTomato-KKT22, endogenous locus, pBA2333	This study	N/A
Plasmid: Inducible GFP-NLS-LacI expression vector, pBA795	(Ishii and Akiyoshi)^42^	N/A
Plasmid: Inducible GFP-NLS-KKT23^2–150^-LacI (KKT23N) expression, pBA2536	This study	N/A
Plasmid: Inducible GFP-NLS-KKT23^125–348^-LacI (KKT23C) expression, pBA2453	This study	N/A
Plasmid: 6HIS-KKT23^2–348^ in pNIC28-Bsa4, pBA1725	This study	N/A
Plasmid: 6HIS-KKT23^125–348^ in pRSFDuet-1, pBA2262	This study	N/A
Plasmid: 6HIS-KKT23^2–70^ in pRSFDuet-1, pBA2426	This study	N/A
Plasmid: 3FLAG-KKT23^2–348^ (codon optimized for expression in Sf9 cells, amplified from BAG111) in pACEBac2, pBA2279	This study	N/A
Plasmid: 3FLAG-KKT23^2–70^ (codon optimized for expression in Sf9 cells) in pACEBac2, pBA2809	This study	N/A
Plasmid: 3FLAG-KKT23^2–149^ (codon optimized for expression in Sf9 cells) in pACEBac2, pBA2810	This study	N/A
Plasmid: 3FLAG-KKT23^71–348^ (codon optimized for expression in Sf9 cells) in pACEBac2, pBA2808	This study	N/A
Plasmid: KKT23^2–348^ (codon optimized for expression in Sf9 cells) in pACEBac2, pBA1994	This study	N/A
Plasmid: 3FLAG-KKT22^2–304^ (codon optimized for expression in Sf9 cells) in pACEBac2, pBA1903	This study	N/A
Plasmid: KKT22^2–304^ (codon optimized for expression in Sf9 cells) in pACEBac2, pBA1904	This study	N/A
Plasmid: 3FLAG-KKT3^2–1058^ in pACEBac2, pBA315	This study	N/A
Plasmid: KKT3^2–1058^ in pACEBac2, pBA882	This study	N/A
Plasmid: KKT3^Δ594–729^ in pACEBac2, pBA2912	This study	N/A
Software and algorithms		
AlphaFold-Multimer	(Jumper et al.; Evans et al.)^46^^,^^47^	https://github.com/google-deepmind/alphafold/releases
ARCIMBOLDO LITE	(Rodríguez et al.)^48^	http://www.ccp4.ac.uk/
ASTRA	Wyatt Technology	https://store.wyatt.com/shop/viscostar/viscostar-iii/astra-software/
BUCCANEER	(Cowtan et al.)^49^	http://www.ccp4.ac.uk/
CCPNmr	(Vranken et al.)^50^	https://www.ccpn.ac.uk
COOT	(Emsley et al.)^51^	http://www2.mrc-lmb.cam.ac.uk/Personal/pemsley/coot/
CRANK2	(Skubak et al.)^52^	http://www.ccp4.ac.uk/
DALI server	(Holm)^19^	http://ekhidna2.biocenter.helsinki.fi/dali/
Diffraction Anisotropy Server	(Strong et al.)^53^	http://services.mbi.ucla.edu/anisoscale/
DisEMBL	(Linding et al.)^54^	http://dis.embl.de
Foldseek	(van Kempen et al.)^20^	https://github.com/steineggerlab/foldseek
HMMER web server	(Potter et al.)^55^	https://www.ebi.ac.uk/Tools/hmmer/
ImageJ	(Schneider et al.)^56^	https://imagej.net
Jalview	(Waterhouse et al.)^57^	http://www.jalview.org/
MAFFT	(Katoh et al.)^58^	https://mafft.cbrc.jp/alignment/server/
MaxQuant	(Cox and Mann)^59^	https://www.maxquant.org/
NMRPipe	(Delaglio et al.)^60^	https://spin.niddk.nih.gov/NMRPipe/
PHASER	(McCoy et al.)^61^	http://www.ccp4.ac.uk/
PHENIX	(Liebschner et al.)^62^	http://www.phenix-online.org/
pLink2	(Chen et al.)^63^	https://www.cog-genomics.org/plink/2.0/
PRIDE database	(Perez-Riverol et al.)^64^	http://www.proteomexchange.org
PyMOL	(DeLano et al.)^14^	http://www.pymol.org/
SEDFIT	(Schuck et al.)^65^	https://sedfitsedphat.github.io/
SEDNTERP	(Hayes)^66^	http://www.jphilo.mailway.com/index.htm
TALOS-N	(Shen et al.)^17^	https://spin.niddk.nih.gov/bax/software/TALOS-N/
TOPSPIN 3.2	Bruker Biospin	https://www.bruker.com/service/support-upgrades/software-downloads/nmr.html
TriTryp database	(Aslett et al.)^67^	https://tritrypdb.org
UniProt	(UniProt)^68^	https://www.uniprot.org
xiView	(Graham et al.)^69^	https://xiview.org/index.php

### Experimental Model and Study Participant Details

#### Bacterial culture

Bacterial strains used in this study are listed in key resources table. Bacterial growth conditions can be found in method details.

#### Cell culture

*Spodoptera frugiperda* Sf9 cells were cultured in SF-900 SFM media (Gibco) at 27° C with shaking (160 rpm).

Procyclic form of *Trypanosoma brucei brucei* cells were cultured in SDM-79 medium supplemented with 10% (v/v) heat-inactivated fetal calf serum.^71^

### Method Details

#### Plasmids

All plasmids used in this study are listed in the key resources table. KKT fragments used in this study were amplified from *Trypanosoma brucei* genomic DNA and cloned into RSFDuet-1 vector (Novagen) for bacterial expression or into pACEBac2 vector (Geneva Biotech) for insect cell expression using NEBuilder HiFi DNA Assembly Kit (New England Biolabs). All constructs were sequence verified.

#### Protein expression and purification

The expression of KKT23^125−348^ and KKT23^2−70^ was carried out as follows. Firstly, *E. coli* BL21(DE3) cells were transformed with ~100 ng of plasmid DNA and then inoculated into 5 mL of 2xTY medium containing 50 μg/mL kanamycin, which was grown overnight at 37° C. For selenomethionine labeling, selenomethionine medium was used instead of 2xTY media (Molecular Dimensions, base mix prepared according to the manufacturer instructions, 5.1 g of nutrients mix and 80 mg of selenomethionine in 1 L of media). In the next morning, each of the 6 L of 2xTY medium with 50 μg/mL of kanamycin was inoculated with 5 mL of the overnight culture and grown at 37 °C with shaking (200 rpm) until the OD_600_ reached ~0.8. Protein expression was induced with 0.2 mM IPTG and was continued for ~16 h at 20°C.

Cells were spun down at 3,400 g at 4°C and resuspended in 200 mL of lysis buffer (50 mM sodium phosphate, pH 7.5, 500 mM NaCl, and 10% glycerol) supplemented with protease inhibitors (20 μg/mL leupeptin, 20 μg/mL pepstatin, 20 μg/mL E−64 and mM PMSF), benzonase nuclease (500 U/l), and 0.5 mM TCEP. All subsequent steps were performed at 4°C. Bacterial cultures were mechanically disrupted using a French press (1 passage at 20,000 psi) and the soluble fraction was separated by centrifugation at 48,000 g for 30 min. Supernatants were loaded onto 5 mL of TALON beads (Takara Bio) pre-equilibrated with lysis buffer. The beads were then washed with ~300 mL of the lysis buffer without protease inhibitors and proteins were eluted with 50 mM sodium phosphate pH 7.5, 500 mM NaCl, 10% glycerol, 250 mM imidazole and 0.5 mM TCEP. To cleave off the His-tag, samples were incubated with TEV protease in 1:50 w/w ratio overnight while being buffer-exchanged into 25 mM sodium phosphate, 250 mM NaCl, 5% glycerol, 5 mM imidazole, and 0.5 mM TCEP by dialysis. The samples were then further purified using ion exchange and size exclusion chromatography. To promote binding of protein to the ion exchange column, the samples were diluted with buffer A (25 mM HEPES pH 7.5 and 0.5 mM TCEP) to achieve the final NaCl concentration of 50 mM. Ion-exchange chromatography was performed using a 6 mL RESOURCE S column for KKT23^125−348^ and a RESOURCE Q for KKT23^2−348^ and KKT23^2−70^, both pre-equilibrated with 5% of buffer B (25 mM HEPES pH 7.5, 1 M NaCl and 0.5 mM TCEP). Protein was eluted with a linear gradient from 5% to 100% of buffer B, concentrated using 10-kD MW Amicon concentrators (Millipore), and loaded on Superdex 75 16/60 (GE Healthcare) columns to further purify and buffer exchange into 25 mM HEPES pH 7.5, 150 mM NaCl with 0.5 mM TCEP. Fractions containing KKT23 were pooled, concentrated and flash-frozen in liquid nitrogen for −80 °C storage.

#### Expression of isotopically labeled KKT23 fragments

The E. coli BL21(DE3) cells were transformed with ~100 μg of plasmid DNA and were inoculated into 40 mL of 2xTY media containing 50 μg/mL kanamycin. Cells were grown at 37°C overnight. The next day, cells were spun down at 3,400 g for 10 min and resuspended in 40 mL of M9 minimal medium containing 50 μg/mL kanamycin, 1 g/L ^15^NH_4_Cl and 4 g/L [^13^C]-D-glucose (CIL) as the sole nitrogen and carbon sources. Next, the resuspended culture was inoculated into 1 L of M9 minimal medium supplemented with 1 g/L ^15^NH_4_Cl, 4 g/L [^13^C]-D-glucose and 50 μg/mL kanamycin. Cells were grown at 37°C to an OD_600_ of 0.9–1.0 and the protein expression was induced using 0.4 mM IPTG. The protein expression was continued overnight at 20° C with shaking (200 rpm).

#### Expression and purification from insect cells

Typically, 50 mL of Sf9 cells at 1–1.2 million cells/mL were infected with 0.5 mL of P3 baculovirus for 72h before harvesting. Cells were pelleted at 700 g for 10 min and resuspended in 2 mL of BH0.25 (25 mM HEPES, pH 8.0, 0.2% NP-40, 2 mM MgCl_2_, 0.1 mM EDTA, 0.5mM EGTA, 10% glycerol, and 250 mM NaCl) supplemented with protease inhibitors (20 mg/mL leupeptin, 20 mg/mL pepstatin, 20 mg/mL E−64, and 0.4 mM PMSF) and 25U of benzonase nuclease. Cells were homogenized on ice by sonication (three rounds of 15 s pulse and 1 min pause) followed by centrifugation for 30 min at 45 000 g. The supernatant was incubated with pre-equilibrated 0.1 mL of anti-FLAG M2 affinity gel (Sigma) for 2.5 h with constant rotation at 4°C. This was followed by five washes with BH0.25 supplemented with 0.5 mM TCEP (1 mL each). To elute the protein, the beads were incubated with 0.1 mL of BH0.25 containing 0.5 mg/mL 3xFLAG peptide (Sigma) for 30 min at RT with gentle agitation. Supernatant containing the eluted protein was separated from the beads using centrifugation and flash frozen in liquid nitrogen for the −70°C storage.

#### Size Exclusion Chromatography with Multi-Angle Light Scattering

MALS experiments were performed during size exclusion chromatography on analytical column SD75 HR10/300 equilibrated with 25 mM HEPES pH 7.5, 150 mM NaCl and 0.5 mM TCEP. Protein elution was monitored via online static light-scattering (DAWN HELEOS 8+, Wyatt Technology), differential refractive index (Optilab T-rEX, Wyatt Technology) and UV (SPD-20A, Shimadzu) detectors. Data were analyzed and molecular weight calculated using the ASTRA software package (Wyatt Technology). KKT23^2−348^ was loaded on size exclusion column at 70 μM in the presence of 140 μM acetyl-CoA.

#### Analytical Ultracentrifugation

The AUC experiments were carried out using a Beckman An-60 Ti rotor in a Beckman Optima XL-1 analytical ultracentrifuge at 20°C. KKT23^2−348^ sample was prepared at 70 μM in the presence of 140 μM acetyl-CoA in 25 mM HEPES pH 7.5, 150 mM NaCl and 0.5 mM TCEP. Scans were recorded using interference optics. Multiple scans were fitted to a continuous size distribution using SEDFIT.^65^ The buffer density and viscosity were calculated with SEDNTERP.^66^ The sample partial specific volumes were estimated from the amino acid sequences, also using SEDNTERP.

#### Crystallization and structure determination

All crystals were obtained in sitting drop vapor diffusion experiments in 96-well plates, using drops of 200 nL overall volume, mixing protein and mother liquor in a 1:1 ratio.

Crystals of *Trypanosoma brucei* KKT23^2–70^ (12.0 mg/mL) were grown at 18°C in ProPlex HT-96 E7 solution (Molecular Dimensions) containing 0.1M Sodium phosphate, pH 6.5, 12% w/v PEG 8000. Crystals appeared after 40 days. Crystals were briefly transferred into mother liquor prepared with addition of 25% glycerol prior to flash-cooling by plunging into liquid nitrogen.

For the native and selenomethionine KKT23^125-348^ samples, before setting the trays, they were supplemented with 1.6 and 0.98 mM acetyl-CoA, respectively. Native crystals of *Trypanosoma brucei* KKT23^125-348^ (14.5 mg/mL) were grown at 4°C in MIDAS HT-96 B9 solution (Molecular Dimensions) containing 0.1 M sodium tartrate dibasic dehydrate, 0.1 M HEPES pH 7.0, 20% SOKOLAN PA 25 CL. Selenomethionine crystals of *Trypanosoma brucei* KKT23^125-348^ (12.5 mg/mL) were grown at 4°C in MIDAS HT-96 F7 solution (Molecular Dimensions) containing 20% v/v jeffamine M-2070, 20% v/v DMSO. Crystals were briefly transferred into mother liquor prepared with addition of 25% glycerol prior to flash-cooling by plunging into liquid nitrogen.

X-ray diffraction data from *Trypanosoma brucei* KKT23^2–70^ were collected at the I04 beamline at the Diamond Light Source (Harwell, UK). The structure was solved using *ab initio* macromolecular phasing software, ARCIMBOLDO LITE^48^ followed by initial model building with BUCCANEER.^49^ Further manual model building and refinement were completed iteratively using COOT^51,72^ and PHENIX.^73^ The datasets used for the final refinement were scaled to the high-resolution limit of 2.7 Å^53^ Electron density for structured water molecules was not observed, therefore, none were modeled.

X-ray diffraction data from selenomethionine crystals of *Trypanosoma brucei* KKT23^125-348^ were collected at the I03 beamline at the Diamond Light Source (Harwell, UK). The structure was solved using CRANK2 software^52^ followed by initial model building with BUCCANEER.^49^ Further manual model building and refinement were completed iteratively using COOT^51,72^ and PHENIX.^73^

X-ray diffraction data from native crystals of *Trypanosoma brucei* KKT23^125-348^ were collected at the I03 beamline at the Diamond Light Source (Harwell, UK). The structure was solved using PHASER^61^ and selenomethionine structure as a model. This was followed by initial model building with BUCCANEER.^49^ Further manual model building and refinement were completed iteratively using COOT^51,72^ and PHENIX.^62^ The dataset used for the final refinement were scaled to the high-resolution limit of 1.75 Å,^53^ followed by two final rounds of refinement using PHENIX.

#### NMR spectroscopy and analysis of NMR data

NMR samples used throughout this study were prepared in 50 mM sodium phosphate pH 7.0 150 mM NaCl, 0.5 mM TCEP and 95% H_2_O/5% D_2_O. KKT23^2–348^ and KKT23^125–348^ were additionally supplemented with 2-fold excess of acetyl-CoA. All NMR spectra were acquired using a 750 MHz spectrometer equipped with a Bruker Avance III HD console and a 5 mm TCI CryoProbe. All NMR data were processed using NMRPipe^60^ and analyzed using CCPN Analysis.^51^ ^1^H, ^13^C and ^15^N chemical shifts of KKT23^125–348^ were analyzed using TALOS-N^16^ to predict secondary structure propensities. The {^1^H}-^15^N heteronuclear NOE was measured for KKT23^2–348^ (0.25 mM + 0.5 mM acetyl-CoA) and KKT23^125–348^ (0.35 mM + 0.7 mM acetyl-CoA) using the TROSY-based heteronuclear NOE experiment recorded with and without ^1^H saturation for 4.0 s at 750 MHz.^74^ The datasets were acquired using 100 complex t1 increments, 88 (KKT23^125–348^) or 116 (KKT23^2–348^) scans per increment and with a ^15^N sweep width of 2283.10 Hz. 1K complex data points were recorded in the F2 dimension with a sweep width of 10638.30 Hz. Data were collected at 20°C for KKT23^2–348^ and KKT23^125-348^. The {^1^H}-^15^N NOE was calculated as the ratio of the peak intensities in the spectra recorded with and without ^1^H saturation. Peak heights were determined using CCPN Analysis.^50^ Uncertainties in the {^1^H}-^15^N NOE values were estimated from 500 Monte Carlo simulations using the baseline noise as a measure of the error in the peak heights.

#### Reconstitution of *T. brucei* and *H. sapiens* nucleosome core particles

Expression and purification of histones, and reconstitution of nucleosome core particles was done as previously described.^27,75^ Briefly, histones were expressed in *E. coli* BL21(DE3)RIL cell inclusion bodies. The inclusion bodies were lysed, sonicated, washed, unfolded, and dialyzed into 7 M urea, 15 mM Tris pH 7.5, 100 mM NaCl, 1 mM EDTA, and 5 mM BME. The lysates were then purified by cation exchange chromatography using a 20 column volume elution gradient of 0–80% 7 M urea, 15 mM Tris pH 7.5, 1 M NaCl, 1 mM EDTA, and 5 mM BME. Protein containing fractions were pooled, dialyzed into 2 mM BME, lyophilized, and stored at −20°C.

Histone octamers were assembled by resuspending the purified, lyophilized histones in 7 M guanidine-HCl, 20 mM Tris pH 7.5, and 10 mM DTT, mixing them at a 1.2:1.2:1:1 (H2A:H2B:H3:H4) molar ratio, and dialyzing the mixture into 15 mM Tris pH 7.5, 2 M NaCl,1 mM EDTA, and 5 mM BME. The resulting complex was purified by size exclusion chromatography (Superdex S200).

Widom 601 145 bp DNA for histone octamer wrapping was isolated from a pUC57 plasmid encoding 8 copies of the 145 bp DNA with EcoRV overhangs using EcoRV restriction enzyme digestion, PEG precipitation, and ethanol precipitation. Nucleosome core particles were then reconstituted by mixing either *T. brucei* or *H. sapiens* histone octamers with the Widom 601 145 bp DNA via a salt dialysis gradient. The nucleosome core particles were subsequently dialyzed into a final storage buffer (15 mM HEPES pH 7.5, 25 mM NaCl, 2.5% glycerol, and 1 mM DTT).

#### *In vitro* histone acetyltransferase assays on reconstituted nucleosomes

Prior to the assay, KKT23^2–348^ was buffer exchanged into the HAT buffer (50 mM Tris-HCl pH 8.0, 50 mM NaCl, 5% glycerol, 1 mM DTT, 0.1 mM EDTA and 10 mM sodium butyrate) using a gel filtration column SD75 16/600. The eluted protein was diluted to 2 μM and 60 μM stocks and flash-frozen in liquid nitrogen for storage at - 70°C. Prior to mixing the enzyme with reconstituted *T. brucei* and human nucleosomes,^27^ acetyl-CoA sodium salt (Sigma Aldrich) was added to the enzyme up to a concentration of 200 μM. The final concentration of the enzyme and substrates was 30 μM and 1 μM, respectively. Then, the enzyme was mixed with the substrate in 1:1 v/v ratio and incubated at 30°C for 1 h. Following incubation, samples were boiled for 3 min and resolved using SDS-PAGE electrophoresis, followed by mass spectrometry (see below) or Western blot analysis. To access composition and quantify loading, samples were resolved using NuPAGE 4–12% gradient gels (Invitrogen) and stained with SimplyBlue (Invitrogen). For the Western blot analysis, proteins from SDS-PAGE gels were transferred onto nitrocellulose membranes, and blocking was done with 5% skimmed milk in PBST. The membranes were then incubated with primary anti pan-acetyl lysine antibodies (#9814S from Cell Signaling) at 1:1000 v/v dilution in PBST overnight at 4°C. Following incubation, the membrane was washed three times with PBST and incubated with anti-rabbit horseradish peroxidase-linked secondary antibodies for 1h at RT (at 1:1000 v/v dilution in PBST). Acetylated proteins were detected by chemiluminescence using SuperSignal West Pico PLUS Chemiluminescent Substrate (Thermo Scientific).

#### Trypanosome cells

Trypanosome cell lines were derived from *T. brucei* SmOxP927 procyclic form cells (TREU 927/4 expressing T7 RNA polymerase and the tetracycline repressor to allow inducible expression.^43^ *Trypanosoma brucei* cell cultures were grown at 28°C in SDM-79 medium supplemented with 10% (v/v) heat-inactivated fetal calf serum.^71^ LacO-LacI tethering experiments were carried out as described previously using the LacO array inserted at the rDNA locus,^34,42^ and expression of GFP-NLS-LacI fusions was induced by the addition of 10 ng/mL doxycycline for 24 h. ChIP-seq analysis was performed as described previously.^4,76^ Briefly, cells expressing KKT3-YFP were fixed with 1% formaldehyde for 20 min at room temperature and sonicated to prepare input chromatin fragments, and KKT3-YFP was immunoprecipitated with rabbit polyclonal anti-GFP antibodies (Thermo Fisher, A11122) that were pre-conjugated with Protein-A magnetic beads (Life Technologies, 10002D), followed by DNA purification. Single-end sequencing (49 bp sequence tag) was carried out on a HiSeq2000 Illumina platform at BGI Hong Kong. ChIP-seq data have been uploaded onto the ENA database (PRJEB76406).

#### Microscopy

Trypanosome cells expressing H2B-GFP or GFP-NLS-LacI fusion with KKT23^2–150^ or KKT23^128–348^ were fixed with formaldehyde as previously described.^5^ Images were captured at room temperature on a Zeiss Axioimager.Z2 microscope (Zeiss) installed with ZEN using a Hamamatsu ORCA-Flash4.0 camera with 63× objective lenses (1.40 NA). Typically, 15 optical slices spaced 0.2 μm apart were collected. Images were analyzed in ImageJ/Fiji.^56^

#### Immunoprecipitation of histones and KKT23^2–150^ from trypanosomes

Initially, a 40 mL culture of procyclic cells expressing either H2B-GFP (BAP2596) or GFP-KKT23^2–150^ (BAP2296) was prepared at a concentration of 1.2 × 10^7^ cells/mL. Subsequently, these cells were inoculated into a larger culture volume of 400 mL at a concentration of 3 × 10^5^ cells/mL, evenly distributed among three flasks. After 24h, KKT23 RNAi-mediated knockdown was induced with doxycycline at 1 μg/mL final concentration in BAP2596. Expression of KKT23^2–150^ in BAP2296 was induced with 10 ng/mL doxycycline. The flasks were then incubated for 1 more day at 27°C until the cells reached approximately 1 × 10^7^ cells/mL. After the incubation period, cells were centrifuged at 800 g for 15 min at room temperature. The supernatant was discarded, and cells were washed with 40 mL of PBS. Following centrifugation at 800*g* for 5 min, cells were resuspended in 25 mL of PEME buffer (100 mM PIPES-NaOH pH 6.9, 2 mM EGTA, 1 mM MgSO_4_, and 0.1 mM EDTA) containing 1% NP-40 and protease inhibitors (Leupeptin, Pepstatin, E-64, 10 μg/mL each, and 0.2 mM PMSF), phosphatase inhibitors (1 mM sodium pyrophosphate, 2 mM Na-beta-glycerophosphate, 0.1 mM Na_3_VO_4_, 5 mM NaF, and 100 nM microcystin-LR), and a histone deacetyltransferase inhibitor (50 mM sodium butyrate). After the 5 min incubation, the lysed cells were centrifuged at 1800 g for 15 min at room temperature. The supernatant was discarded, and the pellet fraction containing the cytoskeleton was transferred on ice and resuspended in 1.5 mL of BH0.15 buffer (25 mM HEPES pH 8.0, 2 mM MgCl_2_, 0.1 mM EDTA pH 8.0, 0.5 mM EGTA pH 8.0, 1% NP-40, 150 mM KCl, and 15% glycerol) supplemented with protease inhibitors (Leupeptin, Pepstatin, E–64, 10 μg/mL each, and 0.2 mM PMSF), phosphatase inhibitors (1 mM sodium pyrophosphate, 2 mM Na-beta-glycerophosphate, 0.1 mM Na_3_VO_4_, 5 mM NaF, and 100 nM microcystin-LR), and a histone deacetyltransferase inhibitor (50 mM sodium butyrate). Samples were then subjected to sonication to solubilize proteins (50% amplitude, 12 s pulse, 60 s pause, 3 cycles). Next, the samples were incubated with 60 μL of Protein-G magnetic beads (Invitrogen) slurry, preconjugated with 12 μg of mouse monoclonal anti-GFP antibodies (Roche, 11814460001) using dimethyl pimelimidate. Following the 2.5 h incubation with constant rotation, the samples were washed with BH0.15 buffer (5 × 1 mL) and eluted with 60 μL of elution buffer (50 mM Tris pH 8.3, 1 mM EDTA and 0.3% SDS) with gentle agitation for 25 min at room temperature. Finally, the samples were frozen and stored at −70°C until further analysis by mass spectrometry.

#### Mass spectrometry: In-solution trypsin digest for immunoprecipitates of H2B-GFP and GFP-KKT23^2–150^ purified from trypanosomes

Reduction of disulphide bridges in cysteine-containing proteins was performed with dithiothreitol (56°C, 30 min, 10 mM in 50 mM HEPES, pH 8.5). Reduced cysteines were alkylated with 2-chloroacetamide (room temperature, in the dark, 30 min, 20 mM in 50 mM HEPES, pH 8.5). Samples were prepared using the SP3 protocol (Hughes et al., 2019) and trypsin (sequencing grade, Promega) was added in an enzyme to protein ratio 1:50 for overnight digestion at 37°C. Next day, peptide recovery in HEPES buffer by collecting supernatant on magnet and combining with second elution wash of beads with HEPES buffer. For further sample clean up an OASIS HLB μElution Plate (Waters) was used. The samples were dissolved in 10 μL of reconstitution buffer (96:4 water: acetonitrile, 1% formic acid and analyzed by LC-MS/MS using a QExactive (GFP-KKT23^2–150^ IP sample) at the proteomics core facility at EMBL Heidelberg, Orbitrap Fusion Lumos mass spectrometer (Thermo) at EMBL Heidelberg (2 replicates of H2B-GFP IP samples), or Orbitrap Fusion Lumos mass spectrometer the Proteomics Facility at Wellcome Center for Cell Biology (1 replicate of H2B-GFP IP samples) as follows.

##### Mass spectrometry data acquisition on QExactive at EMBL (for GFP-KKT23^2–150^ IP)

An UltiMate 3000 RSLC nano LC system (Dionex) fitted with a trapping cartridge (μ-Precolumn C18 PepMap 100, 5μm, 300 μm i.d. x 5 mm, 100 Å) and an analytical column (nanoEase M/Z HSS T3 column 75 μm × 250 mm C18, 1.8 μm, 100 Å, Waters). Trapping was carried out with a constant flow of trapping solution (0.05% trifluoroacetic acid in water) at 30 μL/min onto the trapping column for 6 min. Subsequently, peptides were eluted via the analytical column running solvent A (0.1% formic acid in water) with a constant flow of 0.3 μL/min, with increasing percentage of solvent B (0.1% formic acid in acetonitrile). The outlet of the analytical column was coupled directly to an Orbitrap QExactive plus Mass Spectrometer (Thermo) using the Nanospray Flex ion source in positive ion mode. The peptides were introduced into the QExactive plus via a Pico-Tip Emitter 360 μm OD × 20 μm ID; 10 μm tip (New Objective) and an applied spray voltage of 2.2 kV. The capillary temperature was set at 275°C. Full mass scan was acquired with a mass range 350–1500 m/z in profile mode with resolution of 70000. The filling time was set at a maximum of 50 ms with a limitation of 3 × 10^6^ ions. Data dependent acquisition (DDA) was performed with the resolution of the Orbitrap set to 17500, with a fill time of 120 ms and a limitation of 5 × 10^4^ ions. A normalized collision energy of 30 was applied. Dynamic exclusion time of 30 s was used. The peptide match algorithm was set to ‘preferred’ and charge exclusion ‘unassigned’, charge states 1 and 2 were excluded. MS^2^ data were acquired in centroid mode.

##### Mass spectrometry data acquisition on lumos at EMBL (for 2 replicates of H2B IP experiments)

An UltiMate 3000 RSLC nano LC system (Dionex) equipped with an μ-Precolumn (C18 PepMap 100, 5μm, 300 μm i.d. x 5 mm, 100 Å) and an analytical column (nanoEase M/Z HSS T3 column 75 μm × 250 mm C18, 1.8 μm, 100 Å, Waters) was coupled directly to an Orbitrap Fusion Lumos Tribrid Mass Spectrometer (Thermo) using the Nanospray Flex ion source in positive ion mode. Sample trapping was carried out with a constant flow of 0.05% trifluoroacetic acid in water at 30 μL/min onto the pre-column for 4 min. Subsequently, peptides were eluted via the analytical column running solvent A (0.1% formic acid in water, 3% DMSO) with a constant flow of 0.3 μL/min, with increasing percentage of solvent B (0.1% formic acid in acetonitrile, 3% DMSO) from 2% to 8% in 6 min, to 25% in 41 min, to 40% in 5 min–80% in 4 min and a re-equilibration back to 2% B for 4 min. The peptides were introduced into the Fusion Lumos via a Pico-Tip Emitter 360 μm OD × 20 μm ID; 10 μm tip (CoAnn Technologies) and an applied spray voltage of 2.4 kV. The capillary temperature was set at 275°C. Full mass scan (MS1) was acquired with a mass range 375–1750 m/z in profile mode in the orbitrap with resolution of 120000. The filling time was set at maximum of 50 ms, the AGC target to standard. DDA was performed with the resolution of the Orbitrap set to 15000, with a fill time of 54 ms and an AGC target of 400%. A normalized collision energy of 34 was applied. MS2 data were acquired in profile mode.

##### Mass spectrometry data acquisition on lumos at Edinburgh (for 1 replicate of H2B IP experiments)

Following digestion, samples were cleaned and peptides were concentrated as described by.^77^ Peptides were then eluted in 40 μL of 80% acetonitrile in 0.1% TFA and concentrated down to 1 μL by vacuum centrifugation (Concentrator 5301, Eppendorf, UK). The peptide sample was then prepared for LC-MS/MS analysis by diluting it to 6 μL by 0.1% TFA. LC-MS analyses were performed on an Orbitrap Fusion Lumos Tribrid Mass Spectrometer (Thermo Fisher Scientific, UK) both coupled on-line, to an Ultimate 3000 HPLC (Dionex, Thermo Fisher Scientific, UK). Peptides were separated on a 50 cm (2 μm particle size) EASY-Spray column (Thermo Scientific, UK), which was assembled on an EASY-Spray source (Thermo Scientific, UK) and operated constantly at 50°C. Mobile phase A consisted of 0.1% formic acid in LC-MS grade water and mobile phase B consisted of 80% acetonitrile and 0.1% formic acid. Peptides were loaded onto the column at a flow rate of 0.3 μL min-1 and eluted at a flow rate of 0.25 μL min-1 according to the following gradient: 2 to 40% mobile phase B in 150 min and then to 95% in 11 min. Mobile phase B was retained at 95% for 5 min and returned back to 2% a minute after until the end of the run (190 min). MS1 scans were recorded at 120,000 resolution (scan range 350–1500 m/z) with an ion target of 4 × 10^5^, and injection time of 50ms. MS2 was performed in the ion trap at a rapid scan mode, with ion target of 2 × 10^4^ and HCD fragmentation^78^ with normalized collision energy of 27. The isolation window in the quadrupole was 1.4 Thomson. Only ions with charge between 2 and 6 were selected for MS2. Dynamic exclusion was set at 60 s.

Peptides were identified by searching tandem mass spectrometry spectra against the *T. brucei* protein database with MaxQuant (version 2.0.1) with carbamidomethyl cysteine set as a fixed modification and oxidation (Met), acetylation (Lys), and phosphorylatio006E (Ser and Thr) set as variable modifications. For the analysis of GFP-KKT23^2–150^ sample, up to two missed cleavages were allowed. For the analysis of H2B-GFP samples, up to six missed cleavages were allowed. The first peptide tolerance was set to 10 ppm. To compare acetylation levels on histones in WT and KKT23-depleted cells, intensities of acetylated peptides were normalized by the total amount of each histone protein detected in each sample (Table S2).

#### Mass spectrometry: In-gel digestion for nucleosomes acetylated using recombinant KKT23

The bands corresponding to H2A/H3, H4, or H2B were cut from the gel and subjected to in-gel digestion with trypsin (sequencing grade, Promega). Peptides were extracted from the gel pieces by sonication for 15 min, followed by centrifugation and supernatant collection. A solution of 50:50 water: acetonitrile, 1% formic acid (2 x the volume of the gel pieces) was added for a second extraction and the samples were again sonicated for 15 min, centrifuged and the supernatant pooled with the first extract. The pooled supernatants processed using speed vacuum centrifugation. The samples were dissolved in 10 μL of reconstitution buffer (96:4 water: acetonitrile, 1% formic acid and analyzed by LC-MS/MS using an Orbitrap Fusion Lumos mass spectrometer (Thermo) at the proteomics core facility at EMBL Heidelberg as described above.

Peptides were identified by searching tandem mass spectrometry spectra against the *T. brucei* protein database with MaxQuant (version 2.0.1) with carbamidomethyl cysteine set as a fixed modification, while oxidation (Met) and acetylation (Lys) were set as variable modifications. Up to six missed cleavages were allowed. Total peptide sequence coverages were as follows: 99.3% for H2A, 99.1% for H2B, 99.2% for H3, 99.0% for H4. The first peptide tolerance was set to 10 ppm. We detected "robust acetylation" on K119, K120, K122, K125 and K128 of H2A. These acetylated peptides had MaxQuant intensity higher than 4 3 10^9^, which all had more than 200-fold intensity difference when the corresponding peptides were detected in the control samples (Table S2). In contrast, all other sites had intensity values worse than 4 × 10^8^ and had less than 10-fold difference compared to controls (Table S2).

#### Chemical crosslinking mass spectrometry (XL-MS) of KKT23 complex

Prior to the experiment, bis(sulfosuccinimidyl)suberate, BS^3^ (Thermo Fisher) was equilibrated at room temperature for 2 h and then resuspended to 2.2 mM in distilled water. Immediately after, 2 μL of the crosslinker were mixed with 18 μL of ~3 μM protein complex in 25 mM HEPES pH 8.0, 250 mM NaCl, 10% glycerol, 2 mM MgCl_2_, 0.1 mM EDTA, 0.5 mM EGTA-KOH, 0.1% NP40 and 0.5 mg/mL FLAG peptide resulting final BS^3^ concentration of 220 μM. The crosslinking reaction was incubated on ice for 60 min. To quench the reactions, Tris-HCl at pH 8.0 was added up to 50 mM concentration and the samples were incubated for 10 min at RT. Next, the samples were fresh-frozen in liquid nitrogen and stored at −70°C until mass spectrometry analysis. To estimate the efficiency of the crosslinking reactions, samples were resolved on Nu-PAGE 4–12% gradient polyacrylamide gel (Invitrogen), which was stained using SimplyBlue (Invitrogen). In-solution trypsin digestion with SP3 was carried out as described above at the proteomics core facility at EMBL Heidelberg.

An UltiMate 3000 RSLC nano LC system (Dionex) equipped with an μ-Precolumn (C18 PepMap 100, 5μm, 300 μm i.d. x 5 mm, 100 Å) and an analytical column (nanoEase M/Z HSS T3 column 75 μm × 250 mm C18, 1.8 μm, 100 Å, Waters) was coupled directly to an Orbitrap Fusion Lumos Tribrid Mass Spectrometer (Thermo) using the Nanospray Flex ion source in positive ion mode. Trapping was carried out with a constant flow of 0.05% trifluoroacetic acid in water at 30 μL/min onto the pre-column for 6 min. Subsequently, peptides were eluted via the analytical column running solvent A (0.1% formic acid in water, 3% DMSO) with a constant flow of 0.3 μL/min, with increasing percentage of solvent B (0.1% formic acid in acetonitrile, 3% DMSO) from 2% to 6% in 2 min, to 8% in 2 min, to 30% in 41 min to 40%, in 5 min, to 85% in 7 min, followed by a re-equilibration back to 2% B in 3 min. The peptides were introduced into the Orbitrap Fusion Lumos via a Pico-Tip Emitter 360 μm OD x 20 μm ID; 10 μm tip (New Objective) and an applied spray voltage of 2.4 kV, instrument was operated in positive mode. The capillary temperature was set at 275 C. Full mass scans were acquired for a mass range 350–1700 m/z in profile mode in the orbitrap with resolution of 120000. The filling time was set to auto and the AGC target was set to standard. The instrument was operated in DDA mode and MSMS scans were acquired in the Orbitrap (Resolution at 30000), with a fill time of up to 100 ms and AGC target of 400%. A normalized collision energy of 32 was applied. MS2 data were acquired in profile mode. RAW MS files were searched by the pLink2 software,^63^ with carbamidomethyl cysteine set as a fixed and oxidation (Met) set as variable modifications. Up to two missed cleavages were allowed. Precursor tolerance was set to 10 ppm. All the identified cross-links are shown in Table S4 (FDR 5%). Cross-links were plotted using xiView.^69^ Only crosslinks with a score better than 0.05 are shown in Figure 6.

#### Multiple sequence alignment

The DNA and protein sequences for KKT23 were retrieved from the TriTryp database,^67^ UniProt,^68^ or a published study.^79^ Searches for the homologous proteins were done using BLAST in the TriTryp database^67^ or using hmmsearch using manually prepared hmm profiles (HMMER, version 3.0).^80^ The multiple sequence alignments were performed with MAFFT (L-INS-i method, version 7)^58^ and visualized with the Clustalx coloring scheme in Jalview (version 2.11).^57^

#### *In silico* structure and interaction predictions

Structures and interactions were predicted with AlphaFold-Multimer-v2.3.1^46,47^ through ColabFold version 1.5.5 using MMseqs2 (UniRef+Environmental) and default settings.^81^ The following command was used to map pLDDT score onto the AF2 predicted structure models: spectrum b, rainbow_rev, maximum = 100, minimum = 50.

### Quantification and Statistical Analysis

X-ray crystallography data collection and refinement statistics are summarized in Table 1. Statistical tests and number of replicates are described in the figure legend.

## Supplementary Material

**Supplemental Information**

Supplemental information can be found online at https://doi.org/10.1016/j.str.2024.10.031.

Supplementary Data S1

Supplementary Data S2

Supplementary Figures

Supplementary Table S1

Supplementary Table S2

Supplementary Table S3

Supplementary Table S4

Supplementary Table S5

Supplementary Table S6

## Figures and Tables

**Table 1:** Data collection, refinement statistics

Data collection	*T. brucei* selenomethionine-KKT23^125–348^ (PDB: 9F5Q)	*T. brucei* KKT23^125–348^ (PDB: 9EVQ)	*T. brucei* KKT23^2–70^ (PDB: 9EVR)
Beamline	Diamond Light Source I03	Diamond Light Source I03	Diamond Light Source I04
Wavelength (Å)	0.9786	0.9763	0.9795
Space group (Z)	*P1*	*P1*	*P6_2_22*
Unit cell	42.30 Å 44.53 Å 68.97 Å 100.54° 103.17° 101.33°	42.25 Å 44.42 Å 68.88 Å 100.56° 103.11° 101.24°	57.41 Å 57.41 Å 83.60 Å 90° 90° 120°
Resolution range (Å)^a^	1.95–29.51 (1.95–2.00)	1.63–29.47(1.63–1.67)	2.49–42.73(2.49–2.53)
Unique reflections^a^	32752 (1968)	55472 (3670)	3165 (148)
Completeness (%)^a^	96.6 (82.6)	96.0 (85.7)	100.0 (100.0)
Multiplicity	3.6 (3.4)	3.6 (3.6)	18.4 (19.2)
l/σl^a^	10.4 (2.0)	11.5 (1.8)	12.7 (0.4)
R_meas_	0.110 (0.704)	0.083 (0.741)	0.106 (9.378)
CC_1/2_^a^	1.0 (0.8)	1.0 (0.8)	1.0 (0.7)
Wilson B-factor (Å^2^)	25.62	18.67	58.88
Refinement			
Resolution range (Å)	1.95–29.51	1.75–65.14	2.70–42.73
No. reflections^a^	32733 (1991)	40196 (1537)	2510 (239)
R_work_^a^	0.204 (0.302)	0.178 (0.281)	0.245 (0.476)
R_free_^a^	0.212 (0.333)	0.184 (0.283)	0.265 (0.482)
Number of atoms	3681	4003	386
Protein	3404	3480	386
Ligands	102	102	0
Solvent	175	421	0
RMS bonds (Å)	0.012	0.008	0.010
RMS angles (°)	1.73	1.62	1.09
Ramachandran favored (%)	96.86	97.61	93.48
Ramachandran allowed (%)	2.66	2.39	6.52
Ramachandran outliers (%)	0.48	0.00	2.78
2 Average B-factor (Å^2^)	29.1	24.2	61.26
Protein	29.1	23.6	61.26
Ligands	27.6	20.0	–
Solvent	30.5	29.7	–

a Statistics for the highest-resolution shell are shown in parentheses.

## In brief

Ludzia et al. report the structural and functional analysis of an unconventional kinetoplastid kinetochore protein KKT23. The authors show that KKT23 is a structural homolog of GCN5 histone acetyltransferase that acetylates histone H2A C-terminal tail in trypanosomes. This study also reveals that the N-terminal region of KKT23 interacts with KKT22.

## Highlights

- KKT23 is a structural homolog of GCN5 histone acetyltransferase
- KKT23 acetylates histone H2A C-terminal tail
- KKT23 forms a complex with KKT22 and KKT3 through the N-terminal helical domain

## Resource Availability

### Lead contact

Further information and requests for resources and reagents should be directed to and will be fulfilled by the lead contact, Bungo Akiyoshi (bungo.akiyoshi@ed.ac.uk).

### Materials availability

Plasmids generated in the course of this study can be requested from the lead contact, Bungo Akiyoshi (bungo.akiyoshi@ed.ac.uk). All unique/stable reagents generated in this study are available from the lead contact without restriction.

### Data and code availability

Data generated during this study are included in the manuscript and supplemental information. Protein coordinates have been deposited in the RCSB Protein DataBank (http://www.rcsb.org/) with accession codes PDB: 9EVQ (native *Trypanosoma brucei* KKT23^125−348^), PDB: 9F5Q (selenomethionine *Trypanosoma brucei* KKT23^125−348^), and PDB: 9EVR (native *Trypanosoma brucei* KKT23^2−70^). The chemical shift assignments for KKT23^125−348^ in the presence of acetyl-CoA have been deposited in the BioMagResBank (https://bmrb.io) under the accession number 52461. All raw mass spectrometry files and the custom database files used in this study have been deposited to the ProteomeXchange Consortium via the PRIDE partner repository^64,70^ with the dataset identifiers PRIDE: PXD052922 and PRIDE: PXD052923. ChIP-seq data have been uploaded onto the ENA database (ENA: PRJEB76406). They are publicly available as of the date of publication. Accession numbers are also listed in the key resources table. This paper does not include original code. Any additional information required to reanalyze the data reported in this paper is available from the lead contact upon request.
