# Supplementary material for: The kinetoplastid kinetochore protein KKT23 acetyltransferase is a structural homolog of GCN5 that acetylates the histone H2A C-terminal tail
Source: Structure. Author manuscript; Available in PMC 2026 Oct 15. (PMC7619301; doi:10.1016/j.str.2024.10.031)
Supplement: Supplementary file 1 [file EMS216274-supplement-Supplementary_Figures.pdf]

Structure, Volume *33*

Supplemental Information

The kinetoplastid kinetochore protein KKT23 acetyltransferase is a structural homolog of GCN5 that acetylates the histone H2A C-terminal tail

Patryk Ludzia, Midori Ishii, Gauri Deák, Christos Spanos, Marcus D. Wilson, Christina Redfield, and Bungo Akiyoshi

Figure S1

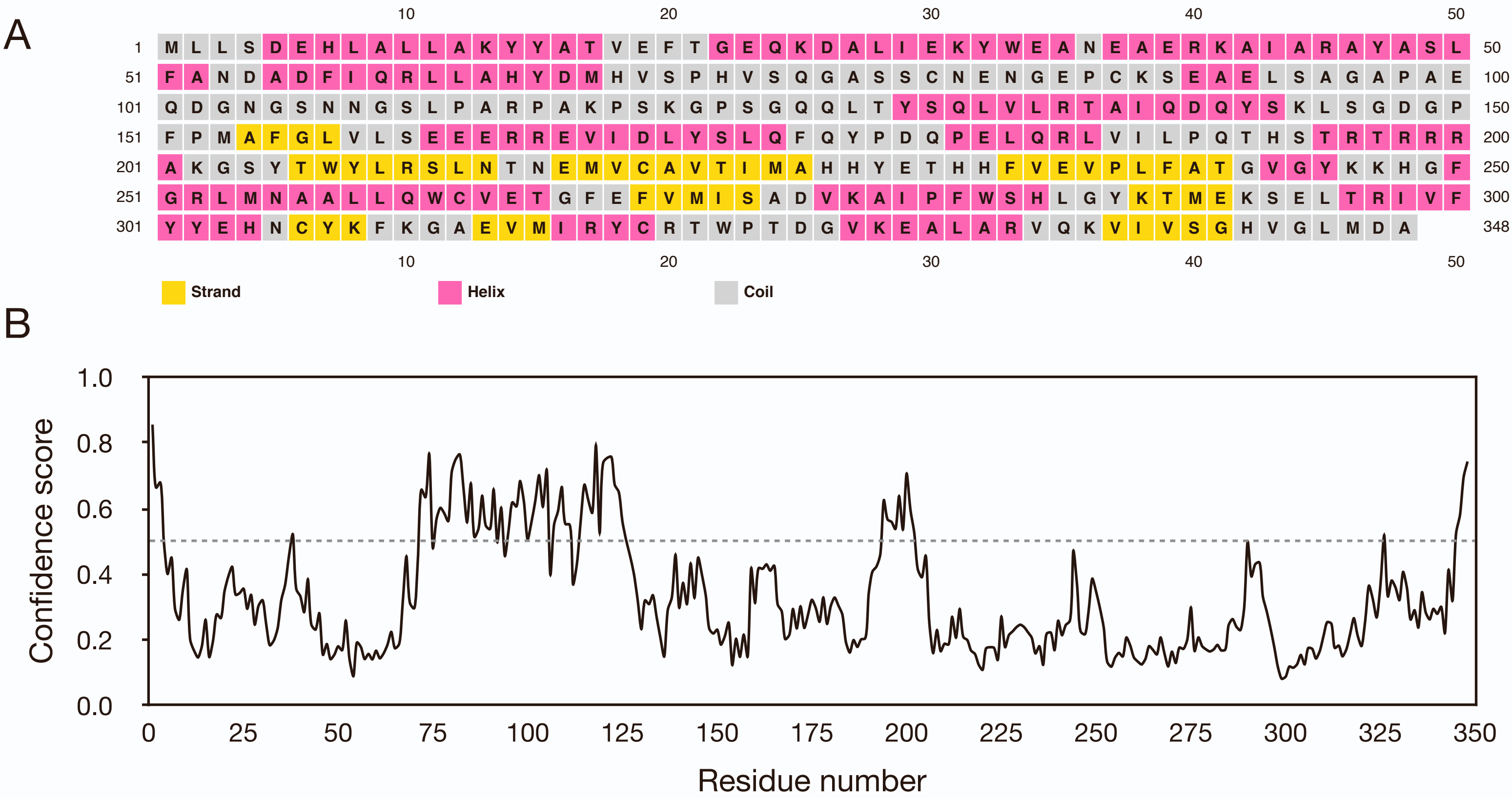

Figure S1. Analysis of the KKT23 primary sequence, related to Figure 1.

A. The secondary structure of KKT23 was analysed using PSIPRED.[1] Each box represents an amino acid labelled with one letter code and coloured according to the predicted secondary structure (yellow for β-strand, pink for α-helix and grey for coil).

B. The disorder probability is plotted against the KKT23 sequence. The prediction was made using the DisEMBL server.[2] The dashed line represents the threshold (0.5) between order and disorder. KKT23 is mainly ordered excluding the regions between 72–126 and 194–202 that have disorder probability values >0.5. This is consistent with the secondary structure prediction shown in (A).

Figure S2

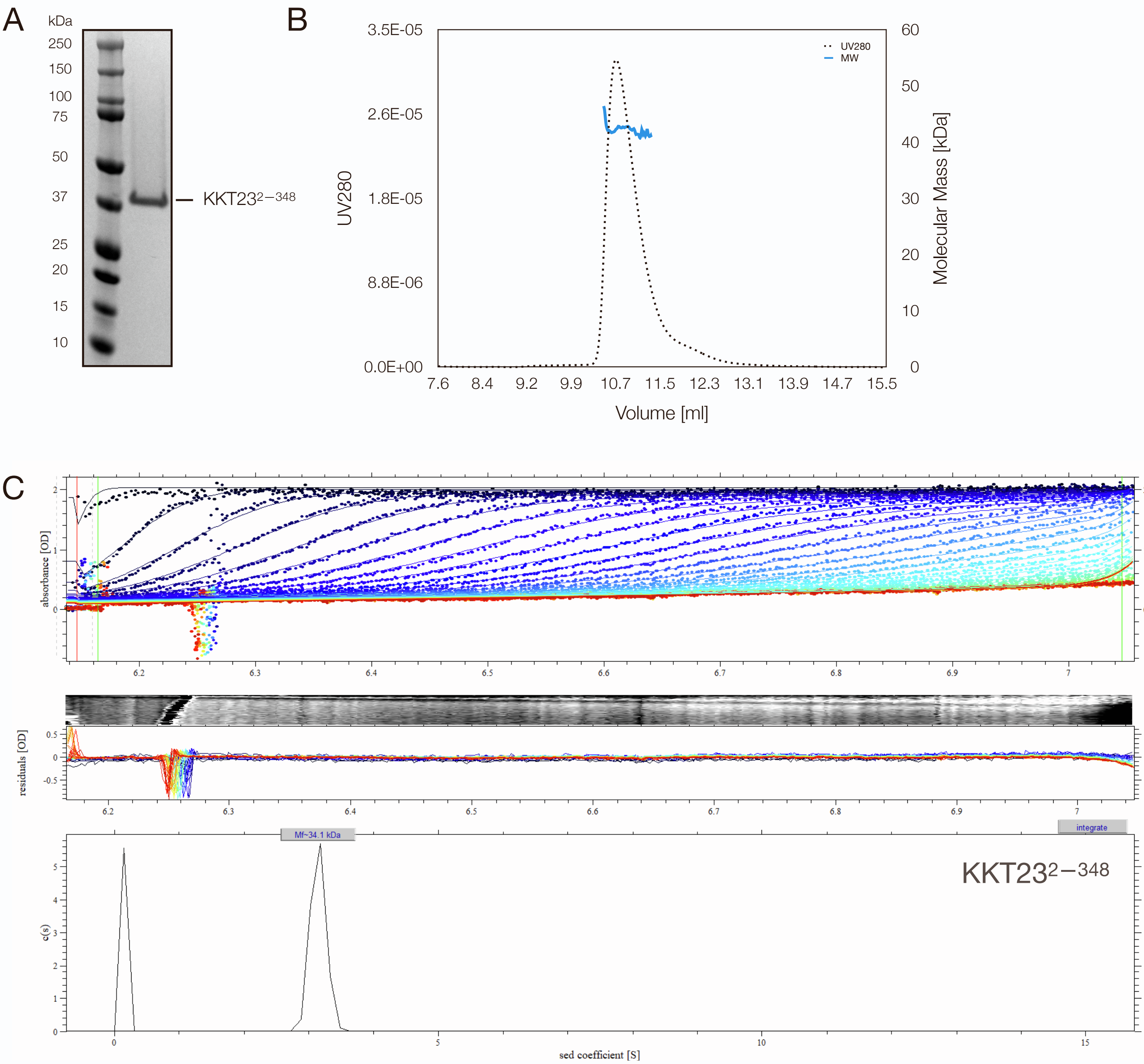

Figure S2. KKT23 is a monomer in solution, related to Figure 1.

A. SDS-PAGE gel stained with Coomassie brilliant blue of the full-length recombinant KKT23. The gel depicts a fraction from size exclusion chromatography run on SD75 16/60 column.

B. SEC-MALS elution profile of 50 μM KKT23$^{2–348}$ in the presence of 100 μM acetyl-CoA. The UV_{280} signal (dotted line) is plotted against the elution volume, while the molecular weight is indicated as a thick line. The estimated molecular weight (~40 kDa) matches the calculated value based on the sequence (39 kDa), suggesting that KKT23 is a monomer.

C. AUC analysis of 50 μM KKT23$^{2–348}$ in the presence of 100 μM acetyl-CoA. The AUC results are consistent with the SEC-MALS analysis in (B), showing that KKT23 sediments as a 34 kDa monomer.

Figure S3

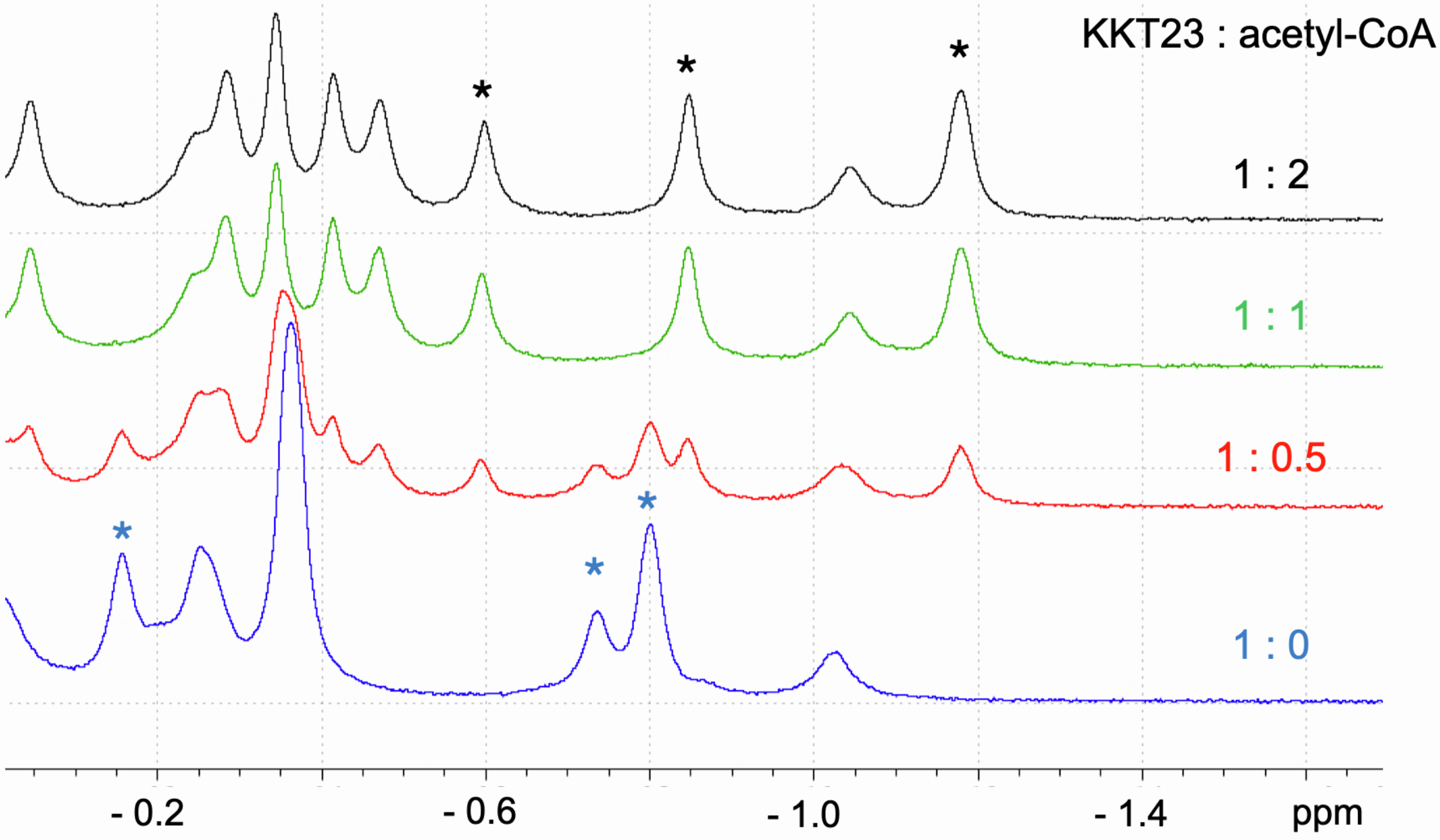

Figure S1. KKT23$^{125–348}$ binds acetyl-CoA, related to Figure 1.

Upfield region of 1D 750 MHz NMR spectra of KKT23$^{125–348}$ confirm the interaction with acetyl-CoA. Spectra of 350 μM KKT23$^{125-348}$ with 0 μM (blue), 175 μM (red), 350 μM (green) and 700 μM (black) of added acetyl-CoA are shown. Blue stars above the 1:0 spectrum indicate apo-KKT23$^{125–348}$ peaks that decrease in intensity as acetyl-CoA is added while black stars above the 1:2 spectrum indicate acetyl-CoA-bound KKT23$^{125-348}$ peaks that increase in intensity as acetyl-CoA is added. KKT23$^{125-348}$ is fully saturated with acetyl-CoA when one equivalent has been added.

Figure S4

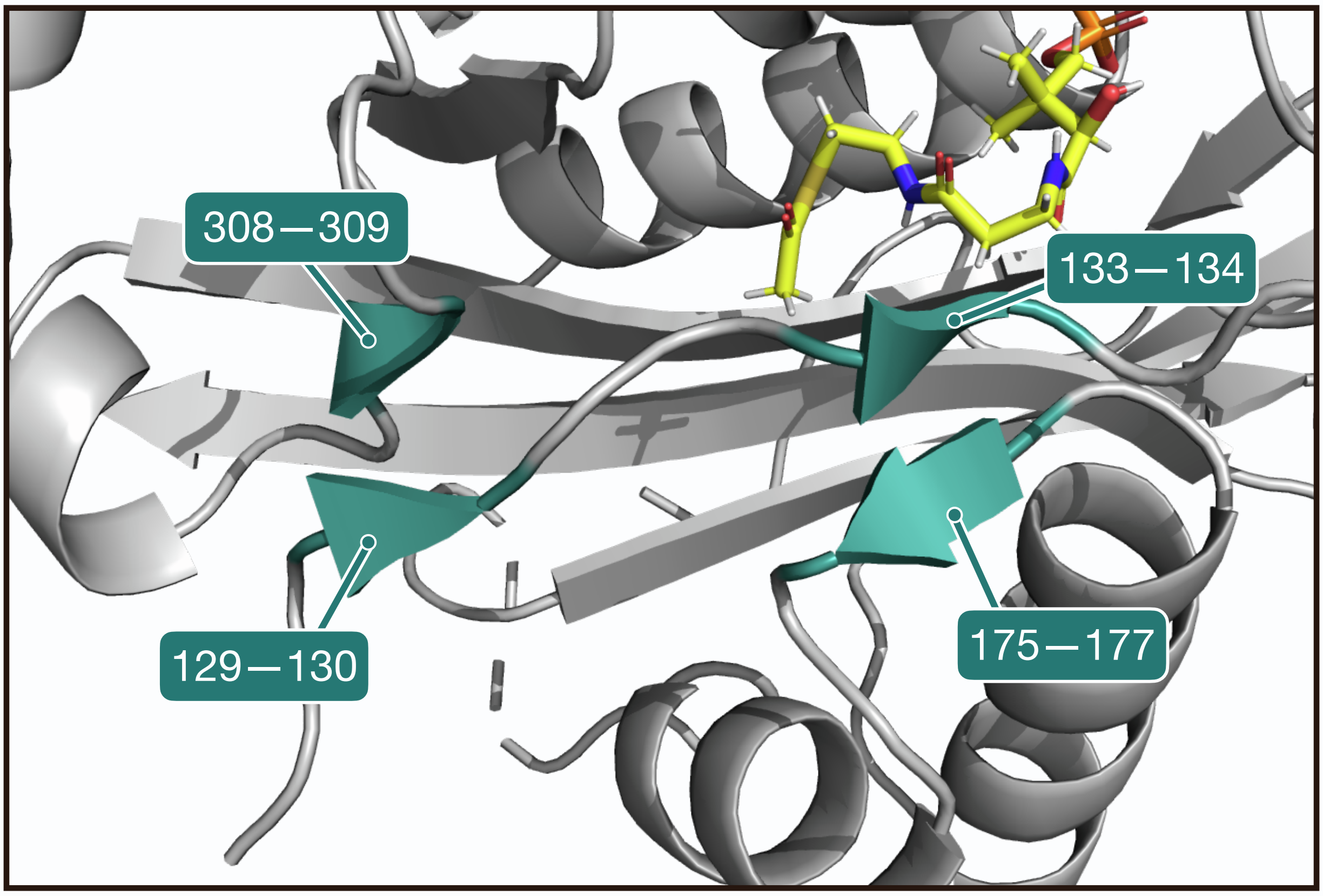

Figure S4. Short β-strands stabilise the unstructured N-terminal region, related to Figures 1 and 3.

In the crystal structure of KKT23$^{125—348}$, four short β-strands were identified to stabilise the N-terminal region by forming β-sheets stabilised by hydrogen bonds. The β-strands involving residues 129–130 and 308–309 were not predicted by TALOS-N; this can be explained by missing assignments for residues 130–132 and 307–310. Peaks from these residues may be broadened, indicating conformational exchange of a slower timescale for this β-sheet in solution. TALOS-N does identify region 133–134 to have β-strand propensity but the 175–177 region is not predicted to be β-sheet. Analysis of phi/psi torsion angles in the X-ray structure shows that residues 133–134 have the expected torsion angles for a β-strand while residues 175–177 do not; regular β-strand torsion angles are required to give rise to the chemical shift values that TALOS-N recognises as arising from β-strand structure. The X-ray structure shows a bifurcated hydrogen bond involving 133 CO and both 176 and 177 N, indicating the distorted nature of this short β-sheet. NOESY spectra show NOEs between 177 HN and 133 Hα and between 133 HN and 175 Hα; these NOEs indicate that in solution residues 133–134 and 175–177 are in close proximity as predicted by the X-ray structure.

Figure S5

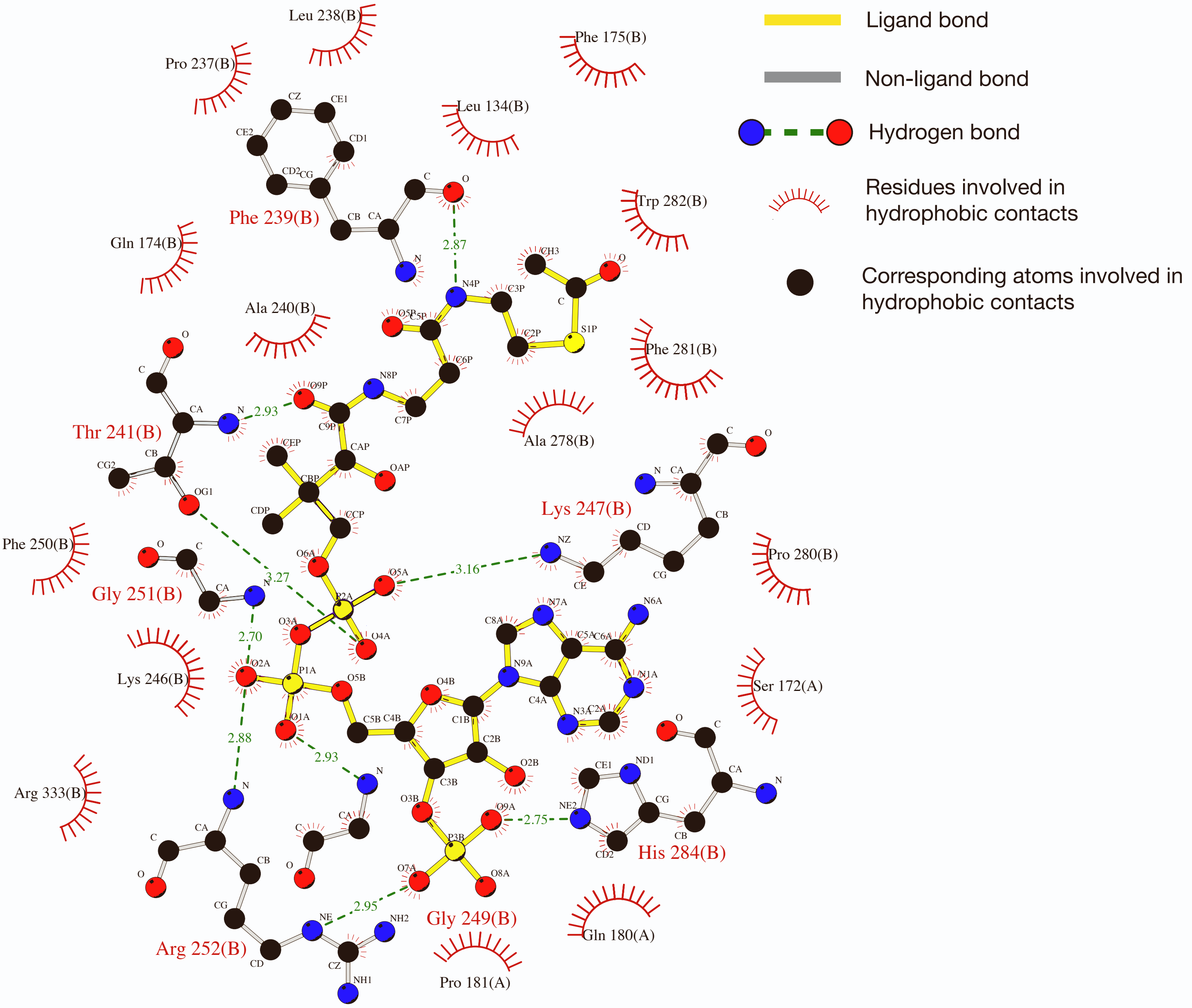

Figure S5. Acetyl coenzyme A is coordinated through hydrogen bonds and hydrophobic interactions in the crystal structure, related to Figure 2.

This figure shows the visual representation of interactions observed between acetyl-CoA and KKT23 residues in the crystal structure of KKT23$^{125–348}$. The acetyl-CoA is coloured in yellow, while the side chains of KKT23 residues are shown as grey sticks. Acetyl-CoA is coordinated through hydrogen bonds and hydrophobic interactions. The figure shows acetyl-CoA bound to molecule B from the crystal structure but also represents the equivalent ligand in molecule A. This figure was prepared and analysed using LIGPLOT[3].

Figure S6

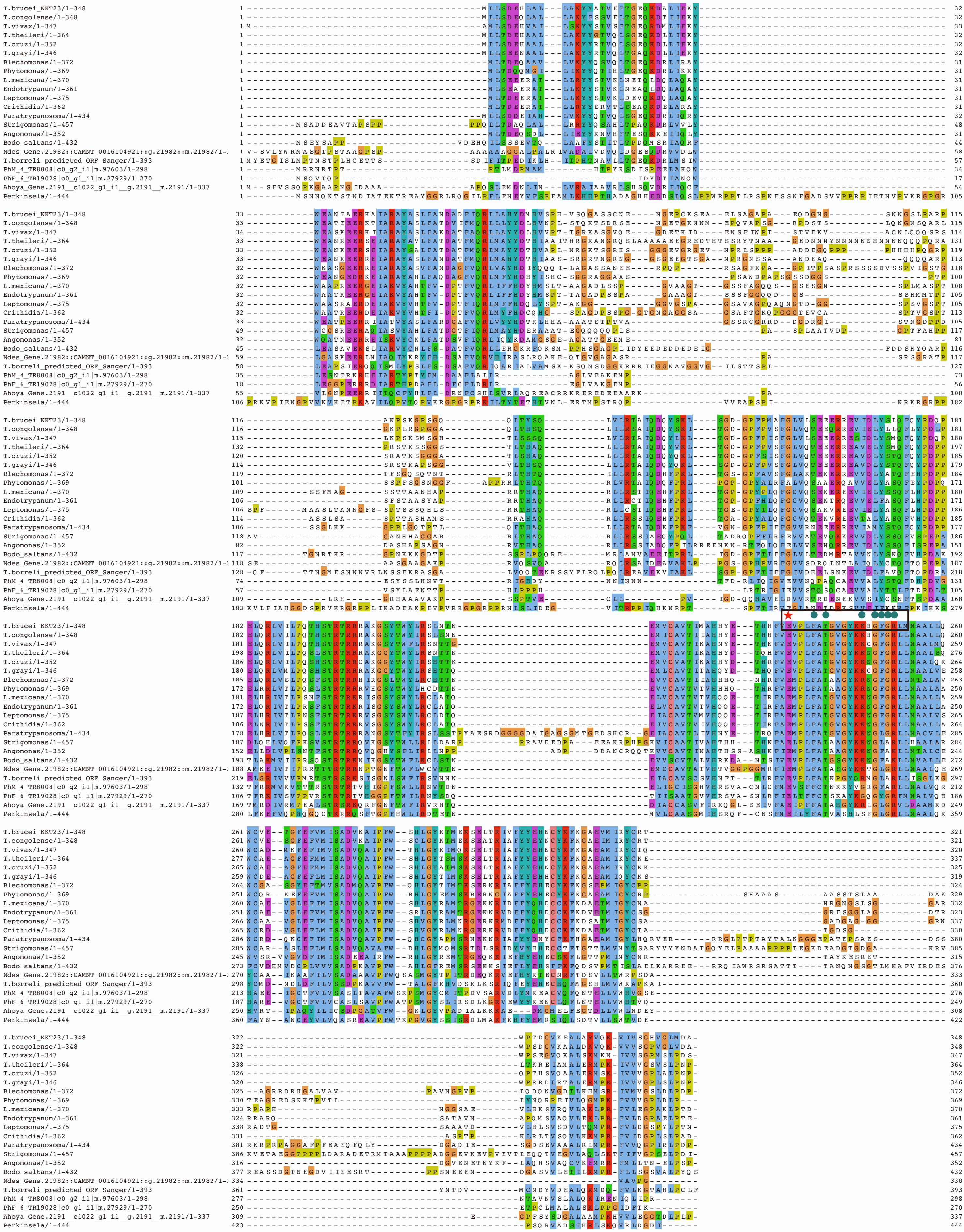

Figure S6. KKT23 is conserved among kinetoplastids, related to Figure 1.

KKT23 has highly conserved protein sequence including the N-terminal helical region and the GNAT domain predicted near the C-terminus (please refer to Figure 1A). The region within 72–126, predicted to be disordered (Figure S1B) is not conserved. The active site and residues that form hydrogen bonds with acetyl-CoA are highlighted with red star and teal circles, respectively.

Figure S7

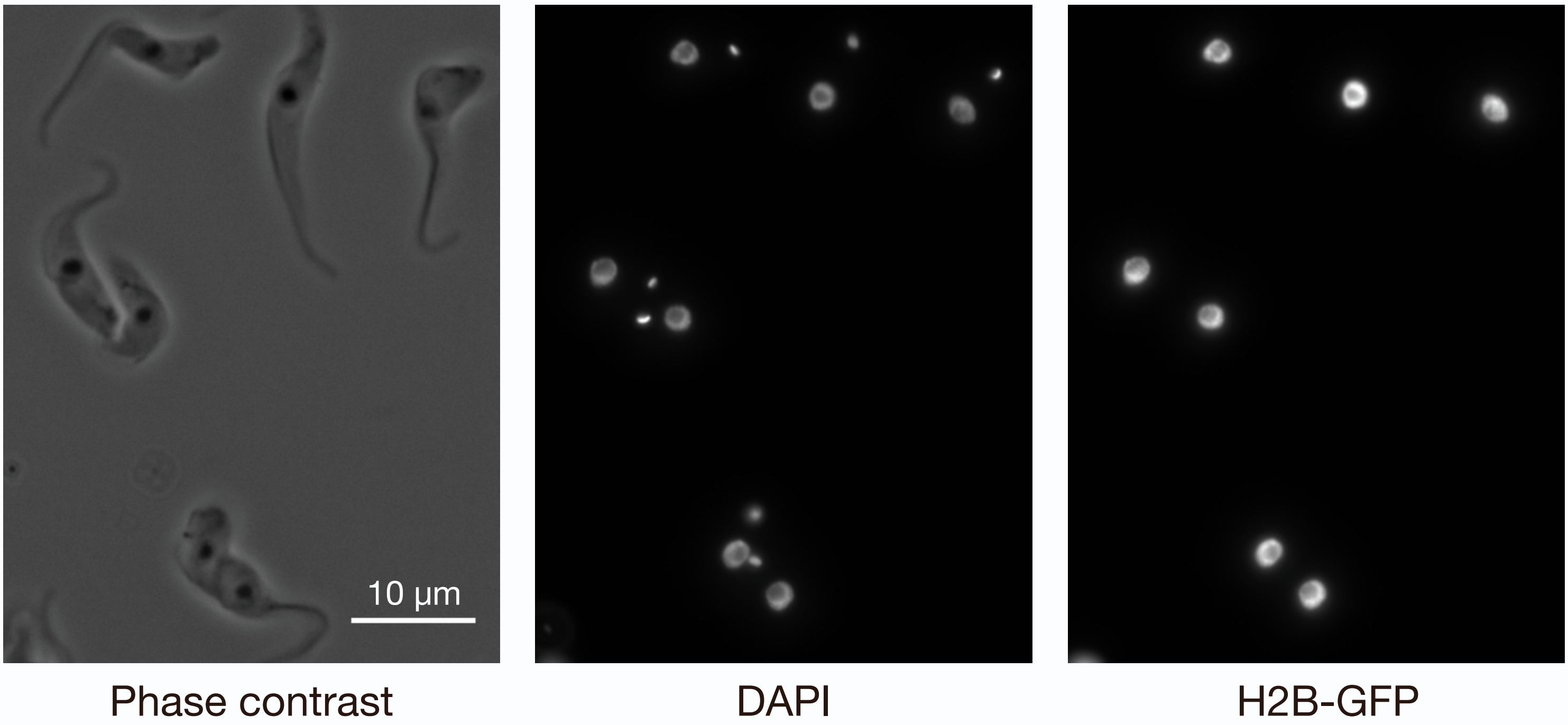

Figure S7. Histone H2B-GFP exhibits nuclear localisation, related to Figure 5.

The left panel presents a phase contrast view of *T. brucei* cells, the middle panel displays DNA staining with DAPI, and the right panel illustrates the nuclear localisation of the endogenous GFP-fused H2B.

Figure S8

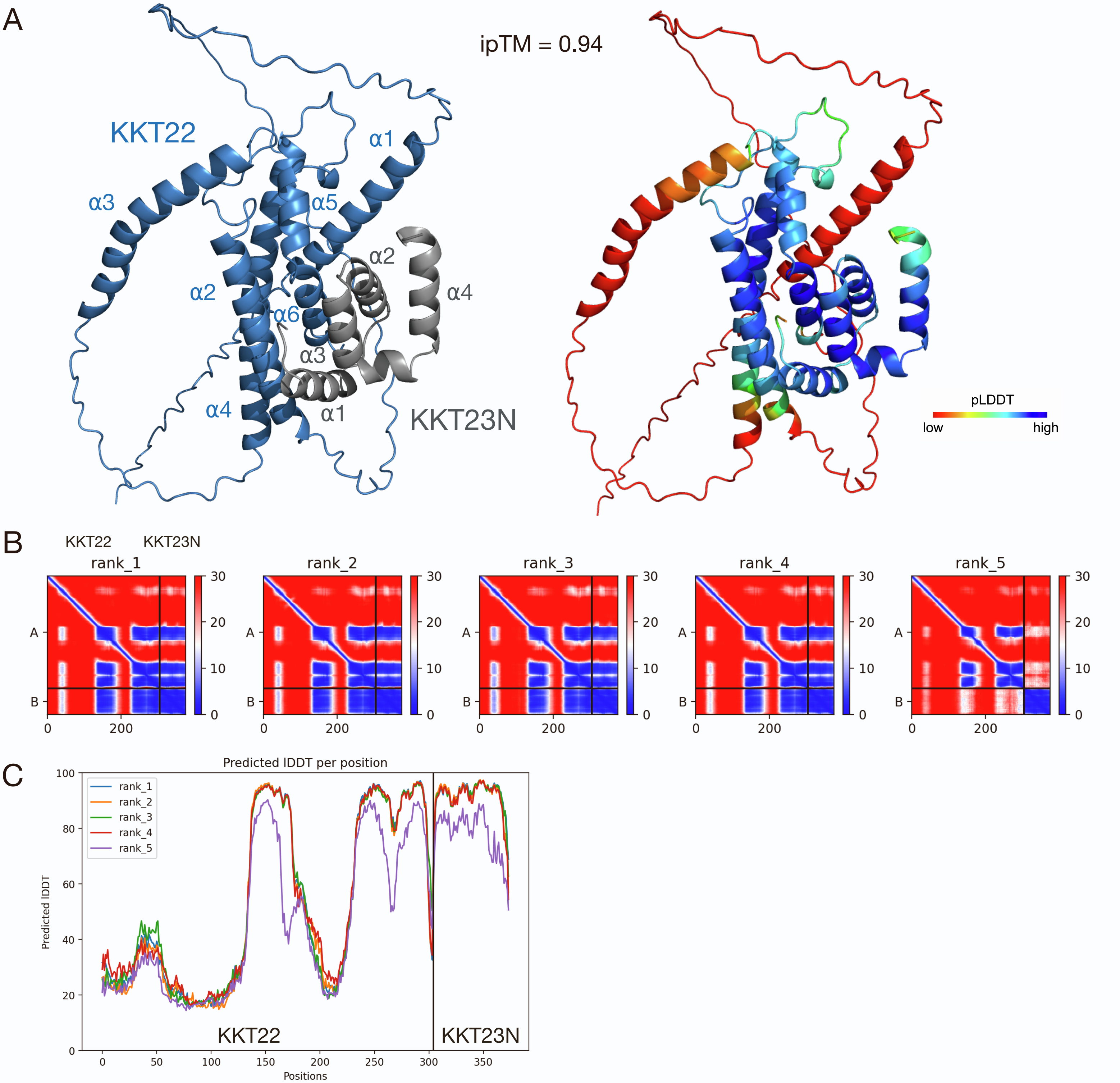

Figure S8. AlphaFold2 predicts interaction between KKT22 and KKT23N, related to Figure 6.

A. AlphaFold2 model (rank 1) of KKT22-KKT23N complex. Cartoon representations are coloured by protein (KKT22 - blue, KKT23N - grey) or according to their pLDDT score. The model was generated over 24 cycles using AF2 colab v1.5.5. The KKT22-KKT23N complex is stabilised by hydrophobic and electrostatic interactions between α2 and α6 on KKT22 and α1–α3 on KKT23N.

B. Predicted Aligned Error (PAE) plots for five models generated by AlphaFold2, coloured by gradient from red to blue. Red and blue indicate high and low error, respectively. The uncertainty in the predicted distance of two amino acids is colour coded from blue (0 Å) to red (30 Å), as shown on the right bar.

C. Predicted Local Distance Test (LDDT) per position for the models (rank 1–5) of KKT22-KKT23N complex, generated by AlphaFold2. Values between 70–90 indicate high accuracy model for the protein backbone.

Figure S9

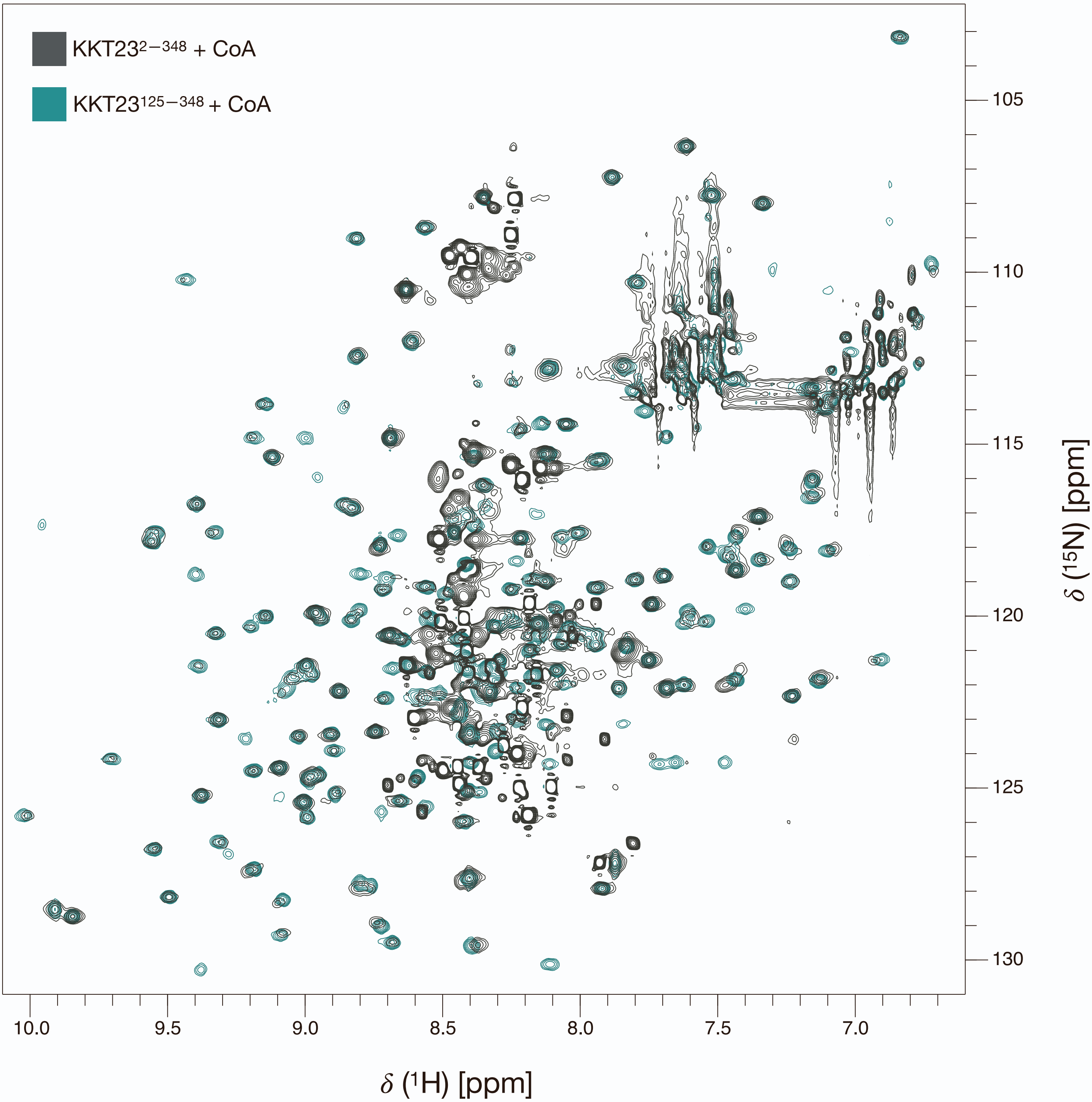

Figure S9. Overlay of spectra obtained for full-length KKT23 and the KKT23 GNAT domain, related to Figure 3.

Overlay of the 750 MHz ^{1}H-^{15}N BEST TROSY spectra of KKT23$^{2–348}$ (grey) and KKT23$^{125–348}$ (teal). Peaks in the spectrum of KKT23$^{125–348}$ overlay well with the spectrum of KKT23$^{2—348}$, indicating that the properties of the GNAT domain are similar in both constructs. Additional peaks in the spectrum of full-length KKT23 located at ~8.0–8.7 ppm have high intensity and are not well dispersed, suggesting that they arise from residues that are disordered. Both spectra were collected using KKT23 samples supplemented with coenzyme A.

Figure S10

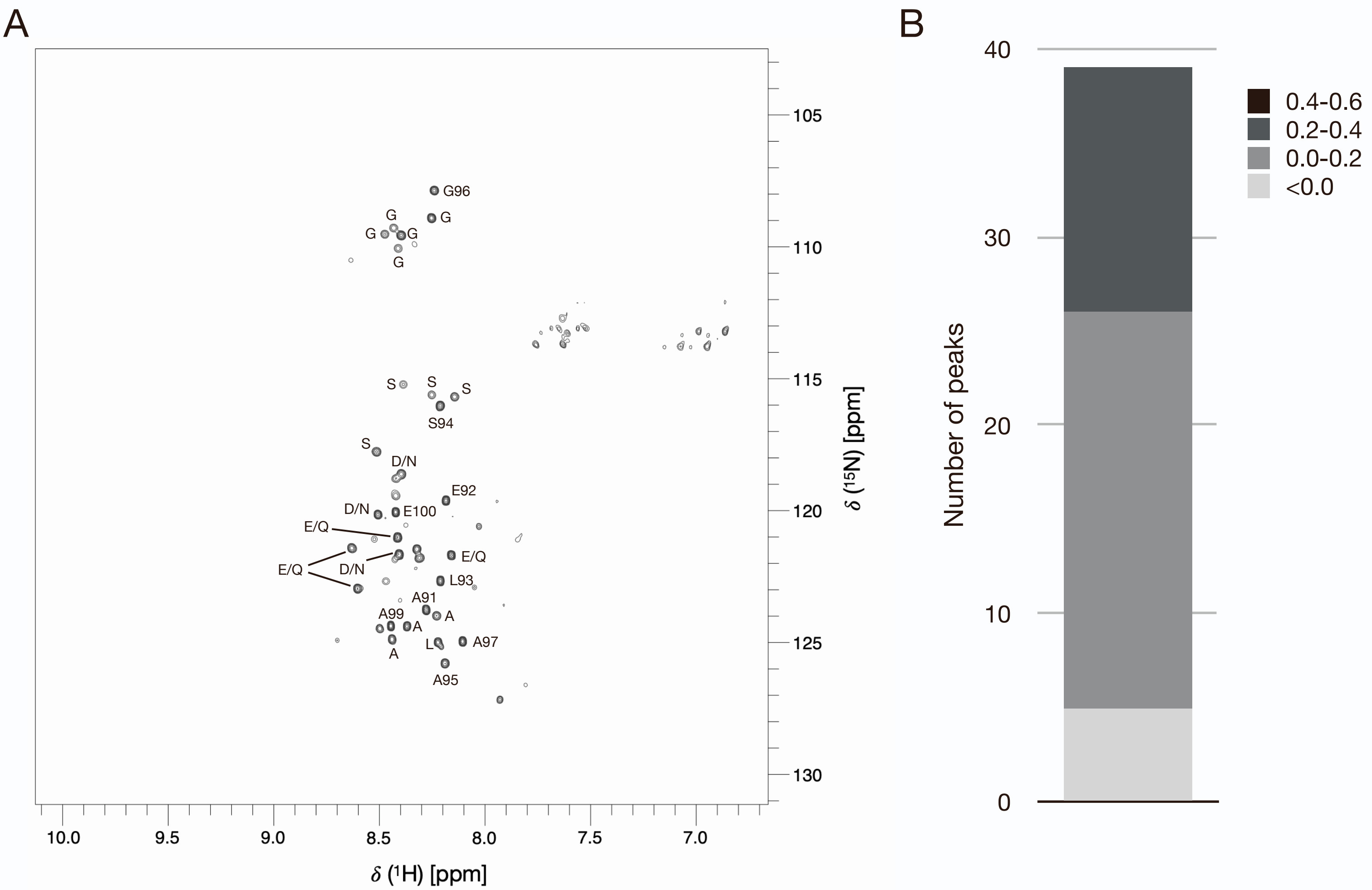

Figure S10. The N-terminal domain and the GNAT domain are connected by a disordered linker, related to Figure 3.

A. The ^{1}H-^{15}N BEST TROSY spectrum of the full-length KKT23 in the presence of coenzyme A was contoured in a way to highlight the high intensity peaks around 8.0–8.7 ppm. These peaks likely correspond to disordered regions of KKT23 predicted between 72–126 (Figure S1B). Some peaks are labelled with sequence-specific residue assignments or with residue-type information. This information was obtained from analysis of 3D 15N-edited TOCSY-HSQC and NOESY-HSQC spectra. The observed Ala, Gly, Leu, Ser, Asp/Asn and Glu/Gln residues are consistent with the low complexity sequence of the linker region of KKT23.

B. The histogram shows the distribution of {^{1}H}-^{15}N heteronuclear NOE ratios measured in KKT4$^{2–348}$ for 39 of the strongest peaks shown in (A). The heteronuclear NOE ratios were segregated and colour coded by different shades of grey according to different values observed. All peaks displayed the hetNOE ratios <0.4, which is characteristic of a flexible backbone. These peaks likely correspond to residues located within the 72–126 region, which is predicted to be disordered (Figure S1B).

Figure S11

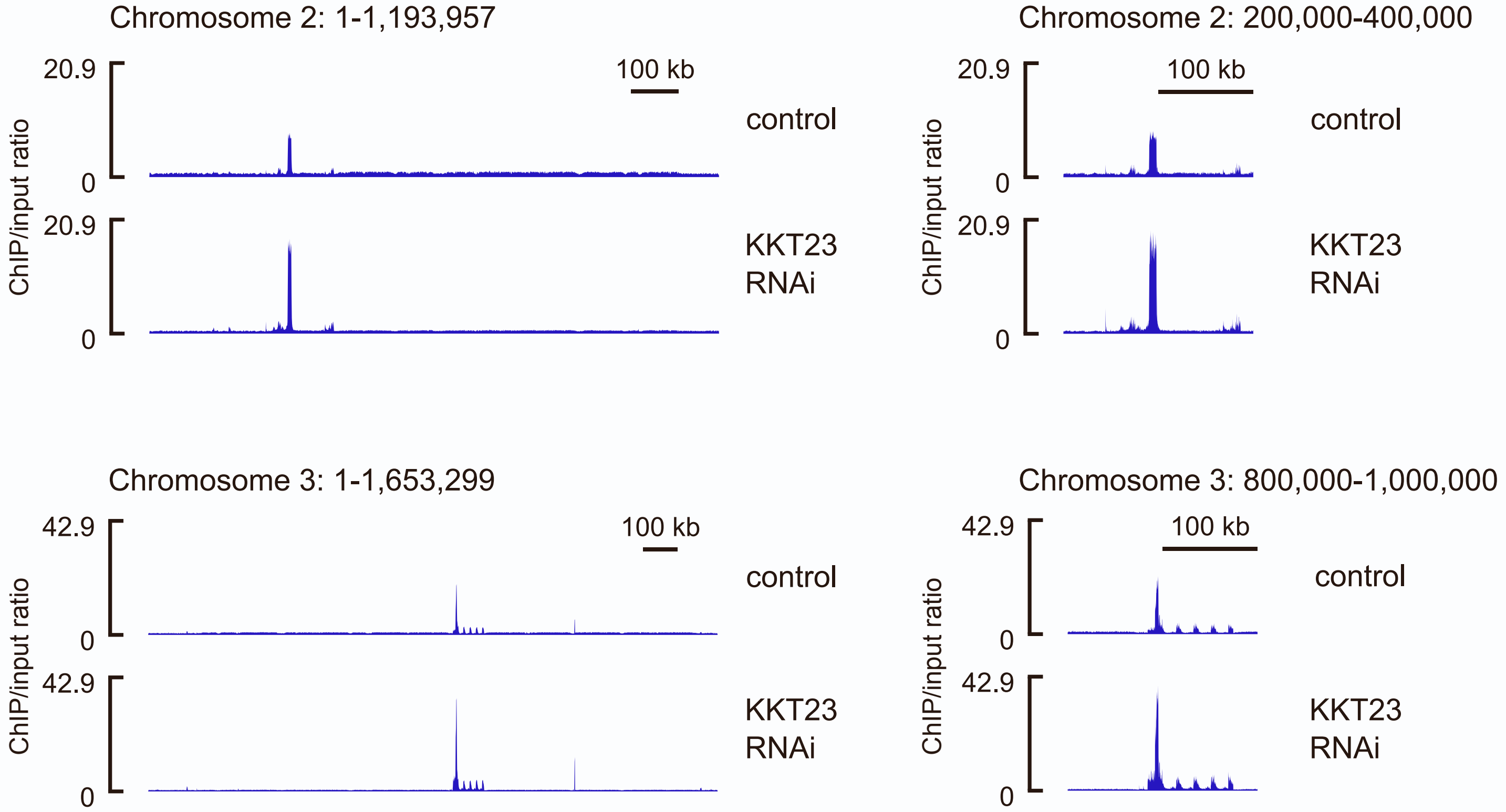

Figure S11. KKT23 depletion does not affect kinetochore position, related to Figure 5.

ChIP-seq data for KKT3-YFP before and after depletion of KKT23 (n=1). The positions in chromosomes where the KKT3 kinetochore protein was enriched did not show any change. RNAi of KKT23 was induced by 1μg/ml doxycycline for 4 days. Data for chromosome 2 (top) and chromosome 3 (bottom) are shown. Cell line, BAP2021

Supplemental references

1. McGuffin, L.J., Bryson, K., and Jones, D.T. (2000). The PSIPRED protein structure prediction server. Bioinformatics. 10.1093/bioinformatics/16.4.404.
2. Linding, R., Jensen, L.J., Diella, F., Bork, P., Gibson, T.J., and Russell, R.B. (2003). Protein disorder prediction: implications for structural proteomics. Structure *11*, 1453-1459. 10.1016/j.str.2003.10.002.
3. Wallace, A.C., Laskowski, R.A., and Thornton, J.M. (1995). LIGPLOT: a program to generate schematic diagrams of protein-ligand interactions. Protein Eng *8*, 127-134. 10.1093/protein/8.2.127.
